# Pan-genomic open reading frames: A potential supplement of single nucleotide polymorphisms in estimation of heritability and genomic prediction

**DOI:** 10.1371/journal.pgen.1008995

**Published:** 2020-08-24

**Authors:** Zhengcao Li, Henner Simianer

**Affiliations:** 1 Animal Breeding and Genetics Group, Center for Integrated Breeding Research, Department of Animal Sciences, University of Goettingen, Goettingen, Germany; 2 State Key Laboratory of Biocontrol, School of Life Sciences, Sun Yat-Sen University, Guangzhou, China; Michigan State University, UNITED STATES

## Abstract

Pan-genomic open reading frames (ORFs) potentially carry protein-coding gene or coding variant information in a population. In this study, we suggest that pan-genomic ORFs are promising to be utilized in estimation of heritability and genomic prediction. A *Saccharomyces cerevisiae* dataset with whole-genome SNPs, pan-genomic ORFs, and the copy numbers of those ORFs is used to test the effectiveness of ORF data as a predictor in three prediction models for 35 traits. Our results show that the ORF-based heritability can capture more genetic effects than SNP-based heritability for all traits. Compared to SNP-based genomic prediction (GBLUP), pan-genomic ORF-based genomic prediction (OBLUP) is distinctly more accurate for all traits, and the predictive abilities on average are more than doubled across all traits. For four traits, the copy number of ORF-based prediction(CBLUP) is more accurate than OBLUP. When using different numbers of isolates in training sets in ORF-based prediction, the predictive abilities for all traits increased as more isolates are added in the training sets, suggesting that with very large training sets the prediction accuracy will be in the range of the square root of the heritability. We conclude that pan-genomic ORFs have the potential to be a supplement of single nucleotide polymorphisms in estimation of heritability and genomic prediction.

## Author summary

The properties of single nucleotide polymorphisms (SNPs) as a main source of genetic variability for estimation of heritability and genomic prediction have been widely studied over the past years. This data type remarkably accelerated the development of medical diagnosis in human genetics and prediction of breeding values in livestock and crop breeding field. However, due to the inherent pitfalls of SNP-based prediction, e.g. imperfect LD between markers and causal variants, seeking new genomic datasets of causal variants has become imperative. Our study point out some of the superiorities of pan-genomic open reading frames as independent variables in estimation of heritability and genomic prediction.

## Introduction

Genome-wide single nucleotide polymorphisms (SNPs) were first proposed in 2001 to be used for predicting genetic values [1]. Implementation in practice became pervasive due to the large amount of SNPs that swiftly became available [2]. By utilizing genome-wide SNP data, ‘genomic selection’ based on genomically predicted breeding values has triggered a revolution in animal and plant breeding. It improved the genetic progress by reducing generation intervals or increasing predictive ability of breeding values [3–5]. In human genetics, genomic prediction aimed at accurately quantifying disease risk so that preventative measures can be taken earlier [6]. However, SNP markers are normally not causal variants. In genomic prediction the causal variant effects are estimated indirectly by modeling SNP makers that are in linkage disequilibrium (LD) with them [2]. The prediction accuracy highly depends on the level of LD between SNP markers and causal variants, and the level of LD depends on the relatedness of the individuals used [7]. For prediction of distantly related individuals, even if high density SNP markers were used, the prediction accuracy still can be very low [8]. Likewise, genome-wide SNP data are also used for estimation or dissection of genetic parameters, such as SNP-based heritability [9]. Several factors inevitably cause the ‘still missing heritability’ problem when using common SNPs exceeding a certain minor allele frequency (MAF) to estimate narrow sense heritability [10]: for instance, the causal variants may not be in complete LD with the SNPs that have been genotyped, or rare variants of large effect are not tagged by common SNPs on genotyping arrays [11, 12]

Pan-genomic open reading frames (ORFs) potentially hold whole-genome protein-coding gene or coding variant information in a population. An ORF is commonly defined as a sequence that has a length divisible by three and begins with a translation start codon and ends at a stop codon. However, a review paper suggests it is bounded by stop codons, since such difinition distinguishes precisely between ORF, exon, and coding sequence (CDS) [13]. An ORF is a sequence region that is ‘open’ for translation, and an indicator for a potential protein-coding gene [13]. The detection of ORFs is of central importance in finding protein-coding genes in genomic sequences. Different individuals may carry partially different sets of genes or ORFs. The ‘pan-genome’ denotes the set of all genes or ORFs present in the genomes of a group of organisms, usually a species [14, 15]. The concept has been applied to bacterial [16], viral [17], plant [18–20], fungal [21], and human genome studies [22]. Series of pan-genomic studies were performed when studying genomic dynamics [23], pathogenesis and drug resistance [24, 25], and species evolution [26].

The budding yeast *Saccharomyces cerevisiae* is a model organism which is not only a premier model for eukaryotic cell biology, but also the pioneer organism for the establishment of the new fields “functional genomics” and “systems biology” [27]. It has previously been shown to be a good tool for exploring the genotype–phenotype relationship via linkage mapping [28], and the study of “missing heritability” [29]. Importantly, *S. cerevisiae* is an informative predictor of human gene function: nearly 50% of human genes implicated in heritable diseases have a yeast homologue [30], which makes *S. cerevisiae* a model species in the studies for prediction of human disease [31]. Structural variants (SVs) such as presence/absence variants (PAVs) and copy number variants (CNVs) have been proven to substantially influence genetic variation and phenotypic diversity [32]. In this study, we used *S. cerevisiae* pan-genomic open reading frames in genomic prediction, which represent 7,796 non-redundant ORFs, accounting either for the presence/absence of a specific ORF or its copy number (CNO). With this we exploited a new source of genome-wide variability for genomic prediction and estimation of heritability, and demonstrated (1) the estimation of heritability based on pan-genomic ORF data and CNO data can capture parts of the “missing heritability” that appears when using SNP data, and (2) genomic prediction capitalizing on ORF data and CNO data performed substantially better than that using genome-wide SNP data.

## Results and discussion

### Population structure based on different genetic variants

**Fig 1:**
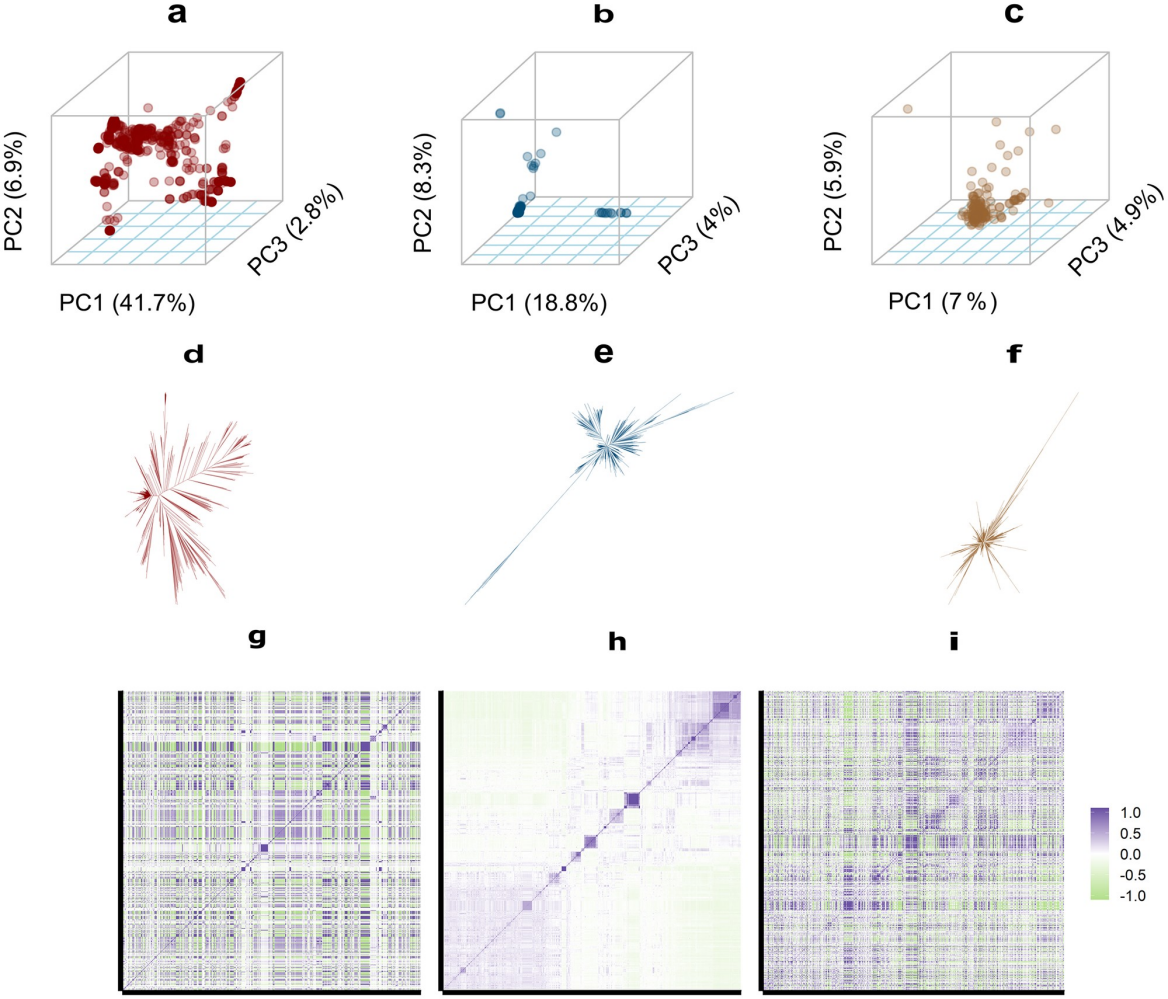
Principal components analysis, neighbor-joining trees and heatmaps of genetic covariance matrices. Panels **a**, **b** and **c** represent principal component (PC) analysis for all common SNPs, pan-genomic ORFs, and the copy numbers of pan-genomic ORFs on 787 diploid *S. cerevisiae* isolates, respectively. PC1, PC2, and PC3 denote the first three principal components. Panels **d**, **e** and **f** represent the neighbor-joining trees of 787 diploid *S. cerevisiae* constructed using the three types of dataset. Panels **g**, **h** and **i** display heatmaps of genetic covariance matrices of 787 diploid *S. cerevisiae* isolates based on all common SNPs, pan-genomic open reading frames, and copy numbers of pan-genomic open reading frames, respectively. Isolates are in the same order in all three panels.

Three types of datasets: all common SNPs, pan-genomic open reading frames, and copy numbers of pan-genomic open reading frames were used for principal components analysis (PCA) on the 787 diploid *S. cerevisiae* isolates [33]. Based on the first three principal components, each type of dataset showed a diverse genetic structure of the *S. cerevisiae* isolates (Fig 1). Compared to the PCA with SNPs where most isolates scattered into a shape of triangle, most isolates in PCA with ORFs and CNOs gathered, but isolates in PCA with CNOs were more scattered than isolates in PCA with ORFs. The first principal component (PC1) in the PCA with SNPs caught 41.7% of the total variance which was much more than PC1 in PCA with ORFs (18.8%) and PCA with CNOs (7%). There are only few isolates for both ORF data which are outliers compared to SNP data, and less than 1% of the phenotypic variance accounted for by either class of ORF genotypes is driven by these outlier strains. When we excluded these outlier strains, the predictive abilities remained the same as with all strains. Analogously, three neighbor-joining trees based on the three types of data were constructed (Fig 1). The ORF-based and CNO-based neighbor-joining trees had similar shapes in which the genetic distances among most isolates were close, and only a few isolates were far away from the other isolates in terms of genetic distance. Five of the ‘outlier’ isolates in ORF-based and CNO-based neighbor-joining trees overlapped. For the SNP-based neighbor-joining tree, the genetic distances among most isolates were relatively large. The heat maps of genetic covariance matrices: *G*, *O*, *C* constructed using the three types of SNP and ORF data are shown in Fig 1, where the yeast strains were in the same order on the basis of their geographical origins in the three matrices. The purple color blocks, indicating high covariance, in the SNP-based genetic covariance matrix were in different positions compared with the purple color blocks in the other two genetic covariance matrices. The purple color blocks in the ORF-based and CNO-based genetic covariance matrices shared similar positions along the diagonal, but compared to the ORF-based genetic covariance matrix, the CNO-based genetic covariance matrix has more blocks indicating high similarity in the off-diagonal regions.

### Capturing “still missing heritability”

**Fig 2:**
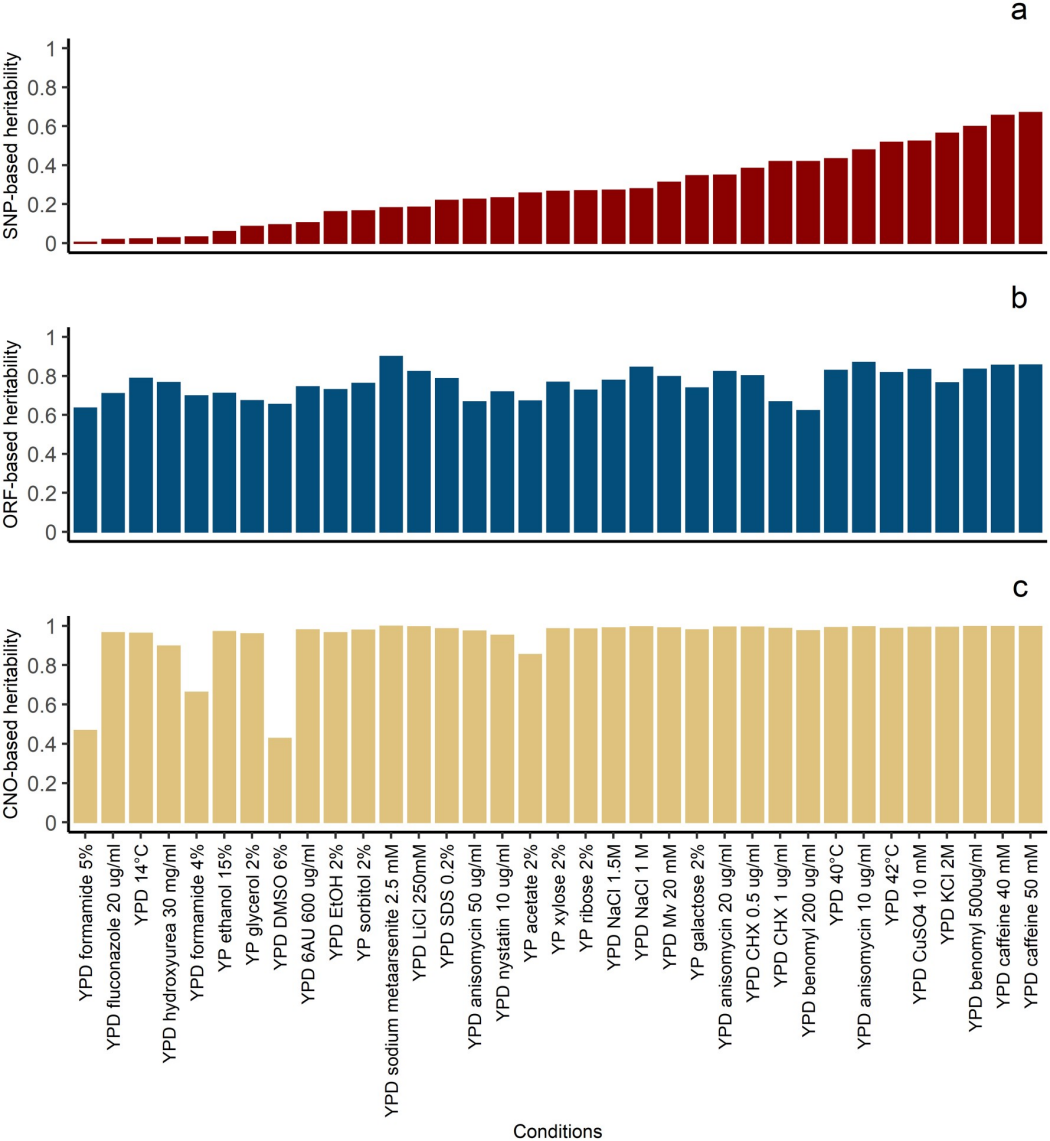
Heritability estimates for all 35 traits estimated based on three data types. Panel **a**, **b**, and **c** depict heritability estimates for all 35 traits estimated based on SNPs with MAF ≥ 0.01, pan-genomic open reading frames with frequency ≥ 0.05, and the copy numbers of pan-genomic open reading frames with frequency ≥ 0.05, respectively. All standard errors were below 0.02.

‘Missing heritability’ has been identified as a critical problem in quantitative genetics: causal variants discovered using genome-wide association studies (GWAS) only explain a small proportion of the phenotypic variation of human height [34]. When using all common SNPs simultaneously in a linear model, 45% of phenotypic variance of human height can be explained, which demonstrated that SNP data without any prefiltering for significance in GWAS could capture a larger part, but still not all of the missing heritability [11]. However, the estimation of SNP-based heritability depended on the extent of LD between SNP markers and causal variants. If SNPs were in low LD with causal variants, which might occur if common SNPs are used but causal variants have low MAF, genomic variants cannot be well tagged by SNPs. Thus, a part of the heritability could still be missing, which was termed “still missing heritability” [10]. we used SNPs with MAF ≥ 0.01, ORFs with frequency ≥ 0.05 and CNOs with frequency ≥ 0.05 to estimate heritabilities, respectively. Our results show that the SNP-based heritability (${\hat{h}}_{G}^{2}$) was 0.281 on average across all traits, ranging from 0.004 to 0.67 (Fig 2 and S1 Table), and the ORF-based heritability (${\hat{h}}_{O}^{2}$) on average across all traits was 0.761, ranging from 0.623 to 0.9, which indicates that pan-genomic ORFs hold more causal variant information than common SNPs in the population. Besides, pan-genomic ORFs were able to capture a major part of the “still missing heritability” for all studied traits, and encompass most of the repertoire of genes or coding variants accessible in the yeast population. This provides evidence that most of the genetic variation of complex traits is additive by nature and can be captured by a linear model [35]. The CNO-based heritability (${\hat{h}}_{C}^{2}$) was 0.935 on average across all traits, ranging from 0.445 to 0.996 (Fig 2), and ${\hat{h}}_{C}^{2}$ captured more “missing heritability” compared with ${\hat{h}}_{O}^{2}$ in 32 of 35 traits. The reason could be that ORF copy numbers reflect a variable number of repeats of some genes with a direct effect on the expression intensity of the related gene product. An example of a complete gene repeat is the copy number polymorphism of human alpha-amylase 1 gene (AMY1), which is directly associated with the amount of salivary amylase and significantly varied between populations with different diets [36]. Another example is the correlation between the copy number of the chemokine gene CCL3L1 and susceptibility to HIV/AIDS, based on significant inter-individual and inter-population differences in the copy number of a segmental duplication encompassing the gene encoding CCL3L1 (MIP-1*α*P) [37]. In addition, ${\hat{h}}_{C}^{2}$ exceeded 0.98 for 20 of the 35 traits, showing that copy numbers of pan-genomic ORFs harbored almost all causal variant information in the yeast population for these traits. However, there were three traits (YPD formamide 5%, YPD formamide 4%, YPD DMSO 6%) for which ${\hat{h}}_{C}^{2}$ was substantially lower than ${\hat{h}}_{O}^{2}$. One possible explanation is that for these three traits ORF repeats were not functional, and thus using the copy number of ORF data presumably added noise in the estimation of genetic variance (S1 Table).

**Fig 3:**
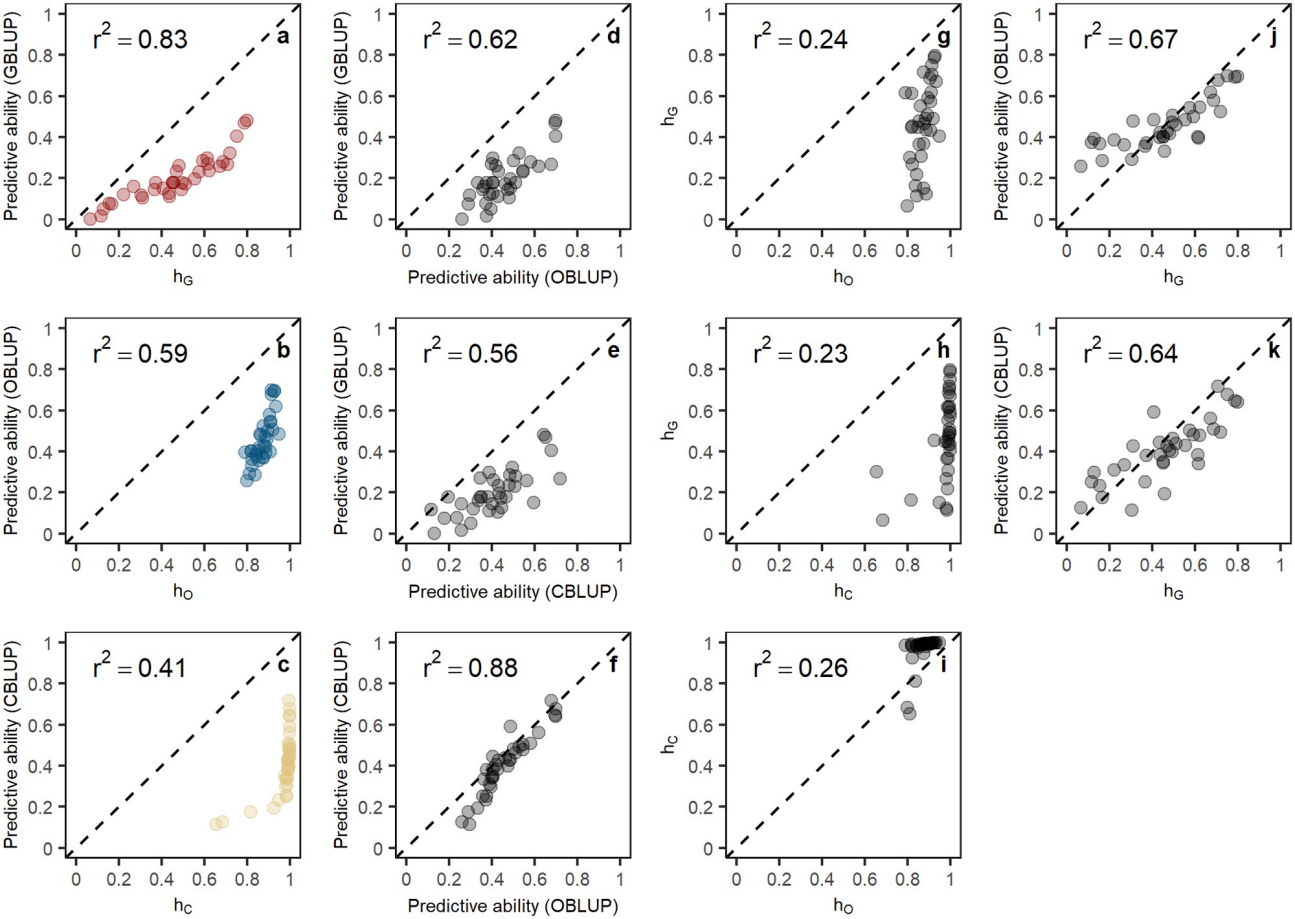
Correlations between predictive abilities and the square root of heritabilities. **(a)** The correlation between predictive abilities of GBLUP and the square root of SNP-based heritabilities across all traits; **(b)** The correlation between predictive abilities of OBLUP and the square root of ORF-based heritabilities across all traits; **(c)** The correlation between predictive abilities of CBLUP and the square root of CNO-based heritabilities across all traits; **(d)** The correlation between predictive abilities of GBLUP and predictive abilities of OBLUP across all traits; **(e)** The correlation between predictive abilities of GBLUP and predictive abilities of CBLUP across all traits; **(f)** The correlation between predictive abilities of CBLUP and predictive abilities of OBLUP across all traits; **(g)** The correlation between the square root of SNP-based heritabilities and the square root of ORF-based heritabilities across all traits; **(h)** The correlation between the square root of SNP-based heritabilities and the square root of CNO-based heritabilities across all traits; **(i)** The correlation between the square root of CNO-based heritabilities and the square root of ORF-based heritabilities across all traits; **(j)** The correlation between predictive abilities of OBLUP and the square root of SNP-based heritabilities across all traits;**(k)** The correlation between predictive abilities of CBLUP and the square root of SNP-based heritabilities across all traits; *h*_*G*_, *h*_*O*_, *h*_*C*_ represent the square root of SNP-based heritabilities, ORF-based heritabilities, CNO-based heritabilities, respectively. *r*^2^ depicts the coefficient of determination. The dots in the 9 panels depict the 35 traits.

When 1’625’809 SNPs were used in estimation of ${\hat{h}}_{G}^{2}$, ${\hat{h}}_{G}^{2}$ increased for 25 traits (S1 Fig), predictive abilities increased in only 8 of 35 traits (S2 Fig). For the remaining 27 traits, the predictive abilities decreased, which suggests that rare SNPs might have caused overestimation of heritability for the majority of traits in this population. This phenomenon is also observed in estimation of ${\hat{h}}_{O}^{2}$. When all ORFs were included, ${\hat{h}}_{O}^{2}$ for all traits increased (S1 Fig), but the predictive abilities for all traits remained the same as with ORFs with frequency ≥ 0.05 (S3 Fig), which suggests that rare ORFs inflate heritability estimates. The square root of ${\hat{h}}_{G}^{2}$ and SNP-based predictive abilities across traits are highly positively correlated. Similarly, for ORF data, the square root of heritability and predictive ability are also remarkably correlated (Fig 3). Nevertheless, the square root of ORF-based heritabilities and ORF-based predictive abilities have a more linear relationship than the result with SNP data (Fig 3), which might be because relationships between training and test set are better explained with ORFs. In addition, the predictive abilities of OBLUP and CBLUP for many traits are higher than the square root of SNP-based heritability (Fig 3). This can be seen as another piece of evidence that ORFs and CNOs capture a part of “missing heritability”. We notice that the values of ${\hat{h}}_{C}^{2}$ for most traits are similar and very high, leaving little room for residuals. To verify whether this phenomenon was caused by rare CNOs, we excluded 1471 CNOs with frequency <0.05. However, the ${\hat{h}}_{C}^{2}$ and CNO-based predictive abilities for all traits remained the same as with all CNOs (S4 Fig), suggesting that rare CNOs are not causal for the apparent bias in heritability estimation. Interestingly, we see “missing” heritability when using SNPs in the model, using ORF or CNO data seems to generate “phantom” heritability, which, however, doesn’t appear to have an adverse effect on predictive ability of the models.

### Improvement of predictive abilities

Precision of SNP-based genomic prediction depends on two factors: SNP-based heritability and the accuracy with which the SNP marker effects are estimated [38]. The square root of the SNP-based heritability provides the upper bound of predictive ability for SNP-based genomic prediction, and this upper bound can be approached when big sample sizes are used for model training [39]. However, the inherent limitation of SNP-based genomic prediction is the extent of LD between SNP markers and causal variants. If causal variants are in low LD with the used set of SNPs, additive genetic effects would be underestimated [11] [40], and the SNP-based heritability would be lower than narrow-sense heritability whose square root is the ultimate upper bound of predictive ability when genetic variance explained by all additive effects is captured. Since there is no perfect LD between causal variants and SNPs, e.g. when rare variants are not captured by common SNPs [10], the ultimate upper bound (narrow-sense heritability) can never be reached when only using SNPs in genomic prediction. Due to this limitation, genomic prediction suffers from diminishing improvements when trying to increase prediction accuracy by increasing the training set size [41]. Thus, it is necessary to explore new sources of predictors to overcome the imperfection.

**Fig 4:**
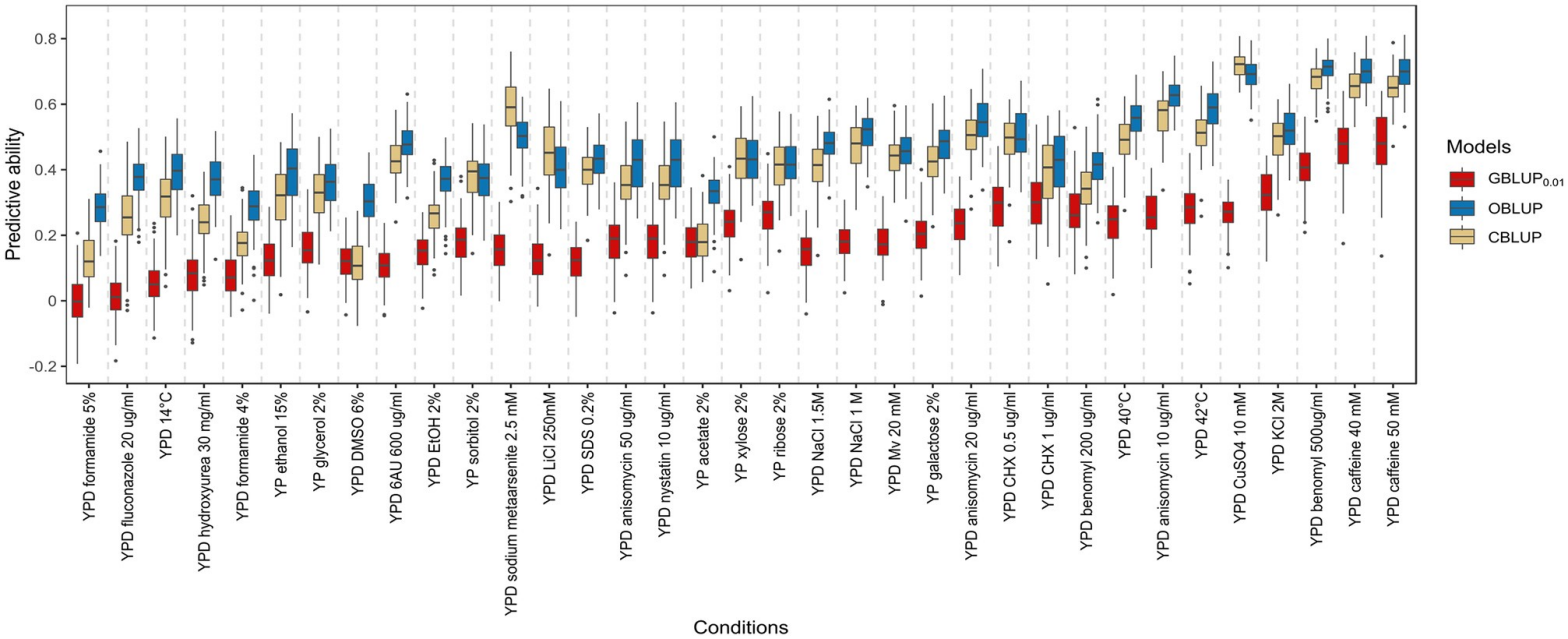
Predictive abilities of three models across 35 traits. GBLUP_0.01_ using all common SNPs, OBLUP using pan-genomic open reading frames, and CBLUP using copy numbers of pan-genomic open reading frames.

**Fig 5:**
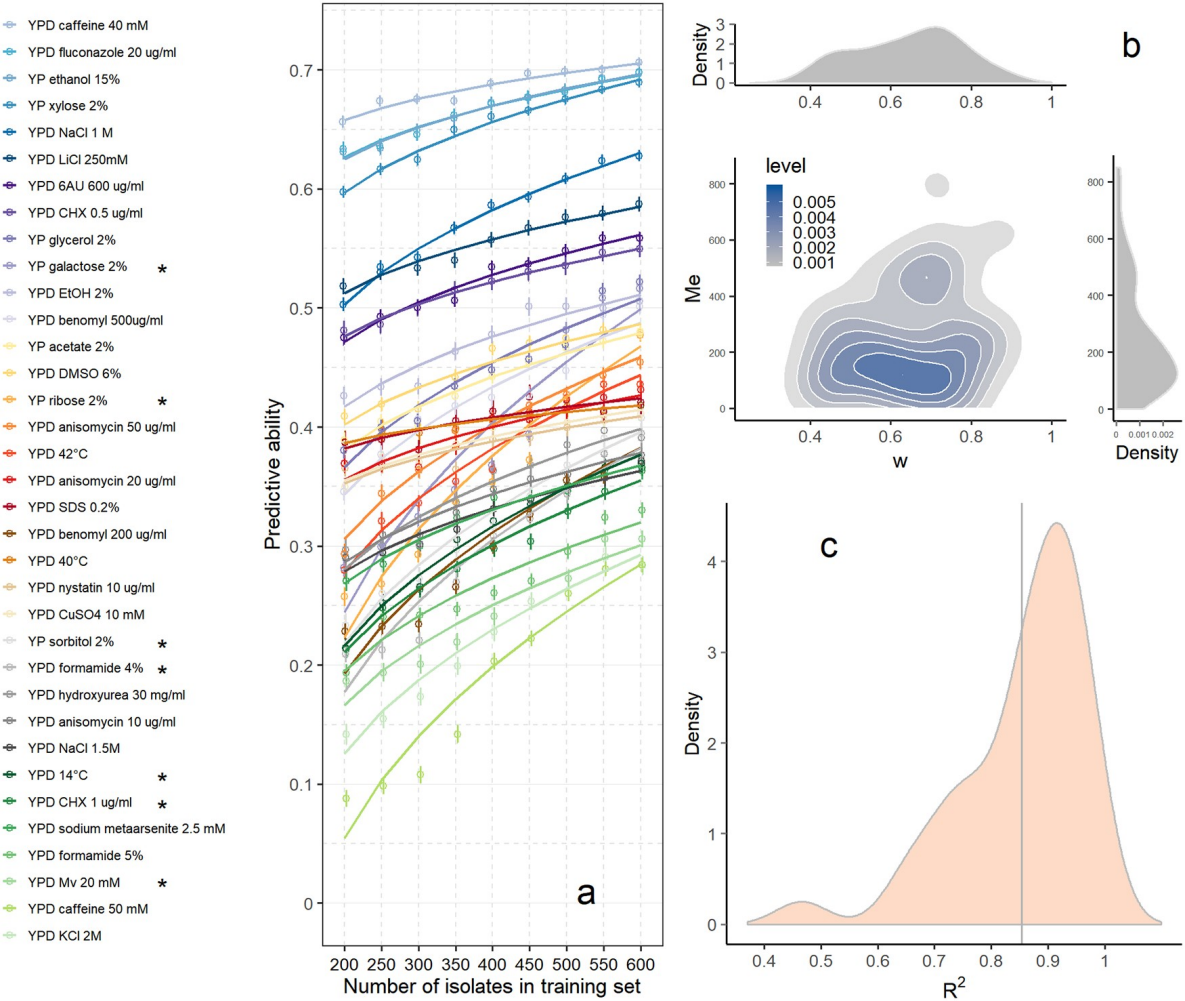
The predictive abilities of ORF-based genomic prediction for 35 traits using different numbers of isolates in training sets. **(a)** When using different numbers of isolates in training sets between *n* = 200 and *n* = 600 in steps of 50 for ORF-based prediction, the predictive abilities of all traits increased as the number of isolates in the training set increased. The solid curves are fitted lines that correspond to the function (2), where r represents the predictive ability in this study (details see text). Traits for which the fitting of the function produced outliers ($\hat{r}>1$) are marked by an asterisk (*). **(b)** The joint distributon of *w* and *Me* for 28 traits with $\hat{r}<1$. **(c)** The *R*^2^ of the fitting of the function (2) for the 35 traits. The vertical line depicts the mean of the *R*^2^ for the 35 traits.

The ‘pan-genome’ denotes the set of all genes or ORFs present in the genomes of a group of organisms [16, 42], which provides an opportunity to accommodate the phenotypic variation caused by the potential protein-coding sequences in a population. We hypothesize that pan-genomic ORFs can be viewed as a representation of a pan-genomic gene set, and using this gene level structure variation set as a supplement of SNPs in genomic prediction will capture more genetic variance than SNP-based prediction. Furthermore, pan-genomic ORFs can also be viewed as a representation of a coding variant set. Causal variants are either coding or regulatory [43]. Coding variants falling within a coding region, especially non-synonymous variants, may change amino acid sequences, and then lead to phenotype variations [44]. In our results, GBLUP as a reference method provided predictive abilities ranging from 0 to 0.48 across the studied traits (Fig 4, S2 Table and S5 Fig). Compared to GBLUP, pan-genomic ORF-based prediction (OBLUP) was more accurate for all traits: observed predictive abilities ranged from 0.28 to 0.71, more than doubled on average across all traits, which manifested the distinct advantage of making use of pan-genomic ORF data in genomic prediction (S6 Fig). When using different numbers of isolates in training sets between *n* = 200 and *n* = 600 in steps of 50 for ORF-based prediction, the predictive abilities of all traits increased as the number of isolates in the training set increased (Fig 5), confirming that increasing the training set size yields more accurately estimated ORF effects. We fitted for each phenotypic trait the function (2): $r=w\sqrt{\frac{n{\hat{h}}_{O}^{2}}{n{\hat{h}}_{O}^{2}+Me}}$ to the ORF data, in which *r* is the observed predictive ability of OBLUP for this trait, *w* is the maximum predictive accuracy with infinite training set size, *n* is the number of isolates in the training set, ${\hat{h}}_{O}^{2}$ is the ORF-based heritability estimate, and *Me* is the number of independent chromosome segments [41]. The parameter *w* is the asymptote of the function given above for *n* → ∞ and thus an extrapolation revealing an estimate of the maximum achievable predictive ability. This model provided an perfect fit of realized prediction accuracies when applied to dairy cattle data [41]. For 28 of the studied traits, the optimal estimate of *w* was in the range of the square root of the heritability of the trait. For seven traits the maximum likelihood optimization yielded estimates of *w* and *Me* which were far beyond the expected range (Fig 5). The distribution of the estimates for *w* and *Me* as well as the correspondence of the estimates of *w* for the 28 traits where the estimates of *r* were <1 are in Fig 5. The medians of the estimates of *w* and *Me* were 0.71 and 293, respectively. When varying the value of the heritability used in ORF-based prediction we observed that this had almost no effect on the prediction accuracy.

It is noteworthy that the pan-genomic ORFs excluded most of non-coding causal variants which are regulatory variants located in non-coding regions. It has been proven that the majority of disease and trait associated variants emerging from genome-wide association analysis studies (GWAS) in humans lie within noncoding sequence that are not in linkage disequilibrium with coding exons [45]. Such non-coding variants may have effects in the gene expression process, such as transcription factor binding, DNA methylation, and mRNA degradation [46], and further influence phenotypes [47, 48]. Nevertheless, when we combined two subsets of total SNP data (MAF ≥ 0.01 and MAF ≥ 0.05) which contained 308’137 SNPs and 102’253 SNPs, respectively, with pan-genomic ORFs in GOBLUP, no more phenotypic variance explained by SNPs was captured, and the predictive abilities remained the same as with OBLUP only using pan-genomic ORF data (S7 Fig), which suggests the noncoding variants have limited impact on the variation of phenotypes in the yeast population, or are not in sufficient LD with the used SNP set. Significantly, the yeast genome is highly compact compared with other eukaryotic species, with ∼70% of the genome sequence comprising ORFs, and a protein-encoding gene can be found for every 2 kb of the genome [49]. By contrast, the human genome contains a potential protein-encoding gene for every 30 kb, and the functional sequence encoding genes covers only ∼30% of the total sequence [50]. The predictive ability of copy numbers of pan-genomic ORF-based prediction (CBLUP) was 0.13 to 0.72, which was significantly higher than the predictive ability of GBLUP (Fig 4 and S8 Fig). For four traits (YP sorbitol 2%, YPD sodium metaarsenite 2.5 mM, YPD LiCl 250mM, YPD CuSO4 10 mM), CBLUP was more accurate than OBLUP, while for the remaining 31 traits, CBLUP was slightly less accurate than OBLUP (Fig 4). The reason could be that some of CNOs were not simple repeats of causal variants, and these CNOs added noise in the prediction. For the second combined method GCBLUP using SNPs and CNOs, the predictive abilities remained the same as with CBLUP for all traits (S9 Fig and S2 Table), suggesting that CNO data covered all causal variant information which SNP data carried. We used two Bayesian methods: Bayes A and Bayes B for predictions with the three types of dataset [1]. Both methods are resampling methods, where in Bayes A marker effects are sampled from a *t*-distribution, while in Bayes B marker effects are assigned a zero effect with probability 1—*π* and are sampled from a *t*-distribution with probability *π* (we used *π* = 0.05). By this, Bayes B accounts for the generally assumed genetic trait architecture, that a limited set of genes, and thus regions in the genome, have an effect on the given complex traits. Difference in predictive abilities between GBLUP and SNP-based Bayes A and Bayes B is negligible in our results. Likewise, when using exclusively pan-genomic ORF data, OBLUP gave similar predictive abilities with ORF-based Bayes A and Bayes B for all traits (S10 Fig), yet Bayes B performed slightly better than CBLUP and Bayes A for 22 traits, when only using copy numbers of pan-genomic ORFs, which indicated that some of the copy numbers of ORF information had no genetic effect (S11 Fig).

Recent pan-genome studies for higher mammals, such as human [22], and pig [51], revealed that non-redundant DNA sequence are absent from current reference genomes, but these studies do not provide ORF information for the populations. In plants, a range of pan-genome studies have shown gene presence/absence variation in many species. Different species present various proportions of core genes: *Brachypodium distachyon* (35%) [2], rice (54%) [52], *Brassica napus* (62%) [53], bread wheat (64.3%) [54], tomato (74.2%) [18], and the proportions decrease when more individuals are added in the pan-genome populations [16]. A *Brassica oleracea* study shows nearly 20% of genes are influenced by presence/absence variation, and some of these genes are annotated with functions related to important agronomic traits, such as flowering time and disease resistance [55]. It has also been demonstrated in the tomato study that such variation may contribute to phenotypic diversity and crop improvement [18]. In yeast *S. cerevisiae* with genome size ∼120Mb about 6’000 genes are reported [49]. Rice (*Oryza sativa L.ssp.indica*) also has a comparatively compact genome (∼460Mb), but still harbours ∼40’000 to ∼50’000 genes, meaning that ∼70% of the rice genome sequence are transcribed in genes [56]. From this angle, one may speculate that pan-genomic ORFs might play a significant role in prediction of rice agronomic traits. Whether pan-genomic ORF data can be used for human disease risk prediction or for animal or plant breeding remains unverified, but one advantage of ORF-based genomic prediction is obvious: ORF-based genomic prediction is not affected by the ‘insufficient LD’ problem which appears in SNP-based estimation of heritability and genomic prediction. Relative to livestock and crops, predicting genotypes or phenotypes using SNPs in humans may be more challenging because the extent of LD in human populations is lower than in domesticated species, which have a long and intensive history of selection and smaller effective population size [57]. In a human genetics’ context, using pan-genomic ORFs as a complement in genomic prediction may have the potential to more accurately identify individuals that are at risk for diseases, and to improve the preventive medicine strategies and clinical decision making. In conclusion, the ORF-based and CNO-based heritability can capture a major part of the “missing heritability”, but we also see that the captured genetic variance is “phantom” to some degree. The ORF-based and CNO-based genomic prediction are more accurate than SNP-based genomic prediction for all traits in the yeast isolates. We demonstrate that pan-genomic ORFs have a potential to supplement SNPs in estimation of heritability and genomic prediction. However, in our study there still is a major gap between heritability and prediction accuracy for all traits, but we provide evidence that prediction accuracy will be further improved if larger sample sizes can be used in training sets.

## Materials and methods

### Whole-Genome SNPs

We used publicly available data from 1,011 *S. cerevisiae* isolates that represent the breadth of their ecological and geographical origins comprised in the 1002 Yeast Genome project. Among these distantly related isolates, 918 had been deep sequenced [33], and the other 93 isolates that had previously been sequenced [58–60]. A total of 1,625,809 high-quality SNPs was reported across the 1,011 genomes. Most of these SNPs were present at very low frequency, with 31.3% of the polymorphic positions being singletons and 93% with a minor allele frequency (MAF) <0.1. After filtering out isolates with aneuploidies, we chose 787 diploid *S. cerevisiae* isolates for which SNP, ORF, copy number of ORF and phenotypes were available for all analyses. We removed SNPs with missing rate >0.05, MAF <0.01, and 311’447 SNPs were remained. 3’310 SNPs which violated Hardy–Weinberg Equilibrium (based on a *χ*^2^ test, p <10^-6^) were also removed. The remaining missing genotypes were imputed using Beagle 4.1 [61]. In total, 308’137 SNPs were used in the analysis. The distribution of minor allele frequency of all common SNPs in 787 diploid *S. cerevisiae* isolates is shown in S12 Fig.

### Pan-genomic open reading frames

The *S. cerevisiae* pan-genome had been determined for the 1,011 genomes using *de novo* genome assemblies and detection of non-reference genome material [33], revealing 7,796 non-redundant ORFs. Among them, 4,940 were core ORFs, containing ORFs present in all isolates and 2,856 ORFs showed a presence/absence variability within the population, containing ORFs that were dispensable or isolate-specific genes. The copy number of each ORF (including copy numbers of core ORFs) was assessed by mapping the reads from each strain to the pan-genomic ORFs with BWA [62]. For details of the *de novo* genome assemblies, detection of non-reference genome material, annotation of ORFs, and the assessment of the ORF copy numbers see [33]. The frequency distribution of pan-genomic open reading frames in 787 diploid *S. cerevisiae* isolates is shown in S12 Fig.

### Phenotypes

Quantitative high-throughput phenotyping had been performed using end-point colony growth on solid medium [33]. 971 strains were phenotyped in parallel under different conditions that affect various physiological and cellular responses. Strains were pregrown in flat-bottom 96-well microplates containing liquid yeast extract peptone dextrose (YPD) medium. Each phenotype value was normalized using the growth ratio between 35 stress conditions and standard YPD medium at 30°C. Pairwise Pearson’s correlations of fitness trait values between replicates were calculated for each condition. In total, 35 fitness traits were used in the present study. The overall statistical description of the 35 traits is shown in S3 Table.

### Statistical models

**GBLUP, OBLUP, and CBLUP**: As a baseline, we conduct the benchmark genomic best linear unbiased prediction (GBLUP) [63], using SNP data. Pan-genomic ORF presence/absence, and copy number of ORF (CNO) information are tested with newly defined approaches termed OBLUP and CBLUP, respectively. The general statistical model is

$$\begin{array}{c} y=1\mu +g+e \end{array}$$ (1)

where *y* is the vector of phenotypic observations, *μ* is the overall mean and 1 is a vector of ones, and *g* ∼ *N*(0, Γ) and $e∼N(0,I\sigma_{e}^{2})$ are vectors containing random additive genetic effects and residual effects, and GBLUP, OBLUP and CBLUP only differ in the covariance matrix Γ used for the genetic effects.

In GBLUP the covariance structure of additive effects was $G\sigma_{g}^{2}$ with $G=\frac{ZZ^{\prime }}{2\sum p_{i}\left(1-p_{i}\right)}$, where *p*_*i*_ denotes the minor allele frequency (MAF) of marker *i*. Moreover, *Z* denotes the MAF adjusted marker matrix with entries (0 − 2*p*_*i*_), (1 − 2*p*_*i*_) and (2 − 2*p*_*i*_) for genotypes 0, 1 and 2, respectively, where the coding refers to the number of reference alleles observed in the genotype.

The ORF-based covariance matrix in OBLUP was calculated as $O\sigma_{o}^{2}$ with $O=\frac{WW^{\prime }}{\sum q_{i}\left(1-q_{i}\right)}$, where *q*_*i*_ denotes the frequency of ORF *i*, and *W* denotes the ORF matrix with entries (0 − *q*_*i*_) and (1 − *q*_*i*_) that represented absence and presence of ORF, respectively.

We fitted for each phenotypic trait the function

$$\begin{array}{c} r=w\sqrt{\frac{n{\hat{h}}_{O}^{2}}{n{\hat{h}}_{O}^{2}+Me}} \end{array}$$ (2)

in which *r* is the observed predictive ability of OBLUP for this trait, *w* is the maximum predictive accuracy with infinite training set size, *n* is the number of isolates in the training set, ${\hat{h}}_{O}^{2}$ is the ORF-based heritability estimate, and *Me* is the number of independent chromosome segments [41]. The two model parameters *w* and *Me* were empirically determined for each of the 35 traits with a maximum likelihood approach using the function “optim” in R [64].

The CNO-based covariance matrix in CBLUP was calculated as $C\sigma_{c}^{2}$ with $C=\frac{SS^{\prime }}{f}$, where *S* denotes the copy numbers of ORFs matrix with entries (*b*_*ij*_ − *u*_*i*_) where 0 ≤ *b_ij_* < 297 represents the copy number of the *i* th ORF in *j* th isolate, and *u*_*i*_ denotes the mean of copy numbers of ORF *i* in all isolates. *f* is a scalar which denotes the median of the diagonal of *SS*′.

Further, we used two models combining SNP and ORF information (GOBLUP), and combining SNP and CNO information (GCBLUP)

$$\begin{array}{c} y=1\mu +g+h+e \end{array}$$ (3)

where $g∼N(0,G\sigma_{g}^{2})$ is a vector containing random additive genetic effects modeled by SNPs, and *h* ∼ *N*(0, *H*) is a vector containing random additive genetic effects where the covariance matrix is derived from pan-genomic ORFs $\left(H=O\sigma_{o}^{2}\right)$ in GOBLUP or from CNOs $\left(H=C\sigma_{c}^{2}\right)$ in GCPLUP, respectively. All other variables are defined as described above.

**SNP, ORF or CNO-based Bayes A and Bayes B**: The model of SNP, ORF or CNO-based Bayes A [1] is

$$\begin{array}{c} y=1\mu +a_{m}+e \end{array}$$ (4)

where *a*_*m*_ is a *m* x 1 vector of normally distributed and independent SNP, ORF or CNO effects. The variance of the *i* th variant effect, $\sigma_{mi}^{2}$ is modeled as a scaled inverted chi-square distribution *χ*^2^ (*v*, *S*), where *S* = 0.002, and *v* = 5. *y*, *μ*, *e* are defined as described above. Gibbs-sampling chains for 50,000 iterations were run, and the first 45,000 burn-in iterations were discarded. The model of SNP, ORF or CNO-based Bayes B [1] is the same as with Bayes A, but the prior distribution of the variance of variant effect is a mixture of distributions which is given by

$$\begin{array}{c} \sigma_{mi}^{2}\{\begin{array}{cc} =0, & \text{with}\;\text{probability}\;\pi \;\\ =\chi^{-2}\left(v,S\right), & \text{with}\;\text{probability}\;\left(1-\pi \right)\; \end{array} \end{array}$$

SNP, ORF or CNO-based Bayes A and Bayes B were implemented in an R package ‘BGLR’ [65].

### Estimation of heritability

The SNP-based heritability was defined as the proportion of phenotypic variance explained by SNP marker effects and calculated as ${\hat{h}}_{G}^{2}=\frac{{\hat{\sigma }}_{g}^{2}}{{\hat{\sigma }}_{g}^{2}+{\hat{\sigma }}_{e}^{2}}$. All SNPs with MAF ≥ 0.01 were used for the estimation [12]. The ORF-based heritability was defined as the proportion of phenotypic variance explained by ORF effects. It was calculated as ${\hat{h}}_{O}^{2}=\frac{{\hat{\sigma }}_{o}^{2}}{{\hat{\sigma }}_{o}^{2}+{\hat{\sigma }}_{e}^{2}}$, using variable ORFs with frequency ≥ 0.05. The copy number of ORF (CNO)-based heritability was defined as the proportion of phenotypic variance explained by the copy number of ORF effects. It was calculated as ${\hat{h}}_{C}^{2}=\frac{{\hat{\sigma }}_{c}^{2}}{{\hat{\sigma }}_{c}^{2}+{\hat{\sigma }}_{e}^{2}}$ using copy numbers of pan-genomic ORFs with frequency ≥ 0.05. Analogously, an ORF-SNP-based heritability ${\hat{h}}_{GO}^{2}=\frac{{\hat{\sigma }}_{g}^{2}+{\hat{\sigma }}_{o}^{2}}{{\hat{\sigma }}_{g}^{2}+{\hat{\sigma }}_{o}^{2}+{\hat{\sigma }}_{e}^{2}}$ and a CNO-SNP-based heritability ${\hat{h}}_{GC}^{2}=\frac{{\hat{\sigma }}_{g}^{2}+{\hat{\sigma }}_{c}^{2}}{{\hat{\sigma }}_{g}^{2}+{\hat{\sigma }}_{c}^{2}+{\hat{\sigma }}_{e}^{2}}$ was calculated. The variance components ${\hat{\sigma }}_{g}^{2}$, ${\hat{\sigma }}_{o}^{2}$, ${\hat{\sigma }}_{c}^{2}$, ${\hat{\sigma }}_{e}^{2}$ from the models above were estimated from the entire data sets, using the R package “regress” [66].

### Comparison of predictive abilities

The predictive abilities of the defined models were measured with 20 replicates of a 5-fold random cross-validation [41]. We defined predictive abilities as the Pearson’s correlation coefficients between predicted genetic values and observed phenotypes in the test sets. The mean of the predictive abilities across 100 estimates was the final predictive ability of each model.

### Principal component analysis

Principal components analysis (PCA) of all common SNPs, pan-genomic open reading frames, and copy number of pan-genomic open reading frames on 787 diploid *S. cerevisiae* isolates was performed using R package ‘factoextra’.

### Genomic and genetic distances

Three neighbor-joining trees were constructed with the R package ‘ape’ using all common SNPs, pan-genomic open reading frames, and copy number of pan-genomic open reading frames, respectively [67]. Isolate dissimilarities were estimated via “Euclidean distance” for each pair of isolates with the “dist.gene” function.

## Supporting information

S1 Fig Heritability estimates for all 35 traits estimated based on all SNPs, all ORFs, and all CNOs, respectively. (TIF)

S2 Fig Predictive abilities of GBLUP_all_, GBLUP_0.01_. GBLUP_all_ using all SNPs, and GBLUP_0.01_ using SNPs with MAF ≥ 0.01.
(TIF)

S3 Fig Predictive abilities of OBLUP_all_, OBLUP_0.01_,and OBLUP_0.05_. OBLUP_all_ using all ORFs, OBLUP_0.01_ using ORFs with frequency ≥ 0.01, and OBLUP_0.05_ using ORFs with frequency ≥ 0.05, respectively.
(TIF)

S4 Fig Predictive abilities of CBLUP_all_, CBLUP_0.01_ and CBLUP_0.05_. CBLUP_all_ using all CNOs, CBLUP_0.01_ using CNOs with frequency ≥ 0.01, and CBLUP_0.05_ usig CNOs with frequency ≥ 0.05, respectively.
(TIF)

S5 Fig Predicted (y-axis) *vs.* observed (x-axis)phenotypes in SNP-based prediction for 35 traits. *r* represents the correlation coefficient between predicted and observed phenotypes across 35 traits.
(TIF)

S6 Fig Predicted (y-axis) *vs.* observed (x-axis)phenotypes in ORF-based prediction for 35 traits. *r* represents the correlation coefficient between predicted and observed phenotypes across 35 traits.
(TIF)

S7 Fig Box plots for predictive abilities of GBLUP_0.01_, GBLUP_0.05_, OBLUP, GOBLUP_0.01_, GOBLUP_0.05_. GBLUP_0.01_ using SNPs with MAF ≥ 0.01, GBLUP_0.05_ using SNPs with MAF ≥ 0.05, OBLUP using pan-genomic open reading frames, GOBLUP_0.01_ using both SNPs with MAF ≥ 0.01 and pan-genomic open reading frames, GOBLUP_0.05_ using both SNPs with MAF ≥ 0.05 and pan-genomic open reading frames.
(TIF)

S8 Fig Predicted (y-axis) *vs.* observed (x-axis)phenotypes in CNO-based prediction for 35 traits. *r* represents the correlation coefficient between predicted and observed phenotypes across 35 traits.
(TIF)

S9 Fig Box plots for predictive abilities of GBLUP_0.01_, CBLUP, GCBLUP_0.01_. GBLUP_0.01_ using SNPs with MAF ≥ 0.01, CBLUP using copy numbers of pan-genomic open reading frames, GCBLUP_0.01_ using both SNPs with MAF ≥ 0.01 and copy numbers of pan-genomic open reading frames.
(TIF)

S10 Fig Box plots for predictive abilities of OBLUP, BayesA_ORF_ and BayesB_ORF_ using pan-genomic open reading frames across 35 traits. (TIF)

S11 Fig Box plots for predictive abilities of CBLUP, BayesA_CNO_ and BayesB_CNO_ using copy numbers of pan-genomic open reading frames across 35 traits. (TIF)

S12 Fig Distribution of minor allele frequency of all common SNPs (red), and distribution of frequency of occurrence of variable ORFs among 787 diploid *S. cerevisiae* isolates (blue). (TIF)

S1 TableHeritabilities estimated from five models across 35 traits.GBLUP, OBLUP, CBLUP, GOBLUP and GCBLUP. ${\hat{h}}_{G}^{2}$ denoted the SNP-based heritability; ${\hat{h}}_{O}^{2}$ the ORF-based heritability; ${\hat{h}}_{C}^{2}$ the CNO-based heritability; ${\hat{h}}_{GO}^{2}$ the SNP-ORF-based heritability; ${\hat{h}}_{GC}^{2}$ the SNP-CNO-based heritability.(PDF)

S2 Table Predictive abilities estimated from five models across 35 traits. GBLUP, OBLUP, CBLUP, GOBLUP and GCBLUP.
(PDF)

S3 Table Statistical description of phenotype data. (PDF)
