# Supplementary figures and images for: Pan-genomic open reading frames: A potential supplement of single nucleotide polymorphisms in estimation of heritability and genomic prediction
Source: PLoS Genet. 2020 Aug 24;16(8):e1008995. doi: 10.1371/journal.pgen.1008995 (PMC7470747; doi:10.1371/journal.pgen.1008995)

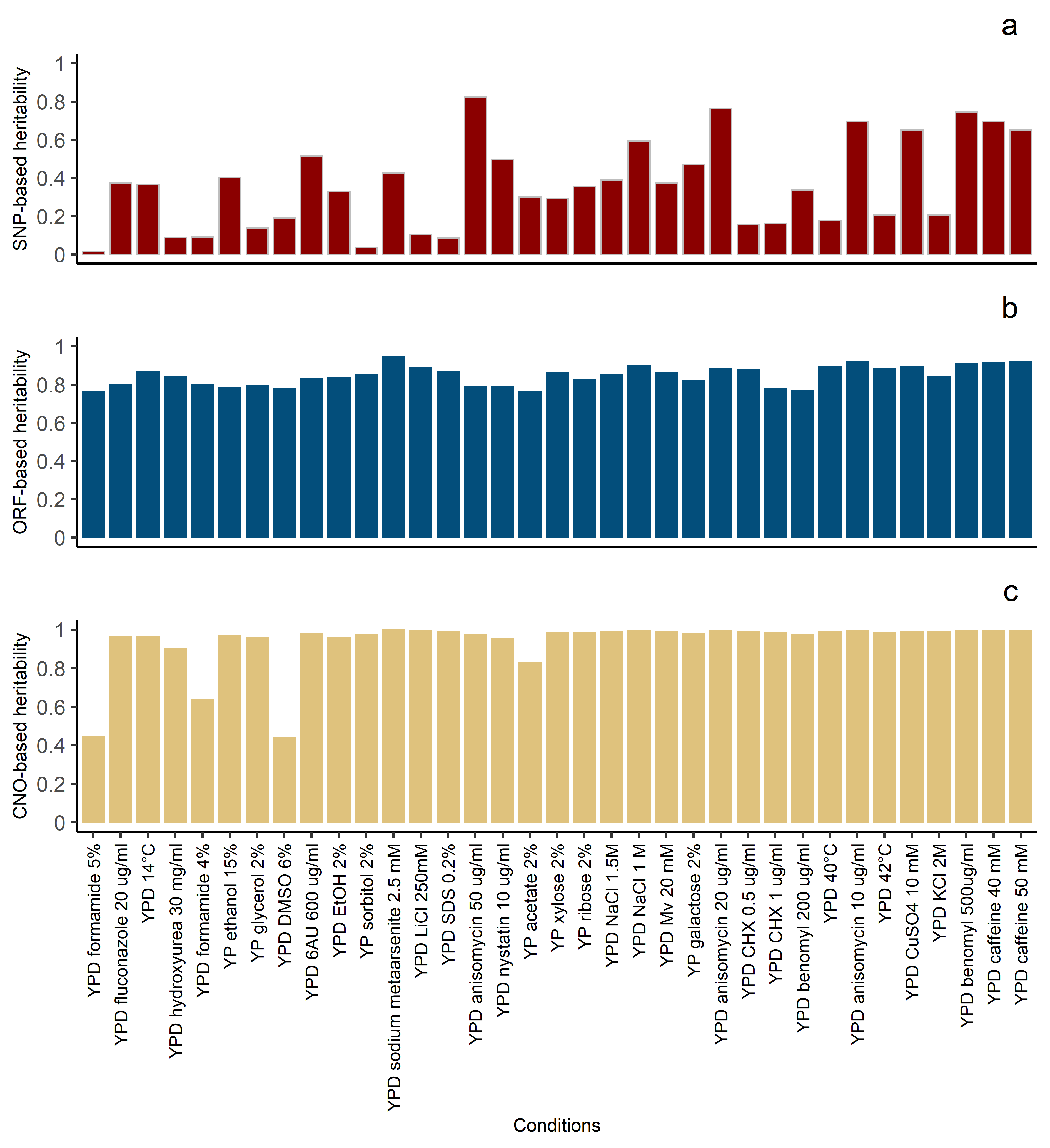

Supplement: S1 Fig — (TIF) [file pgen.1008995.s001.tif]

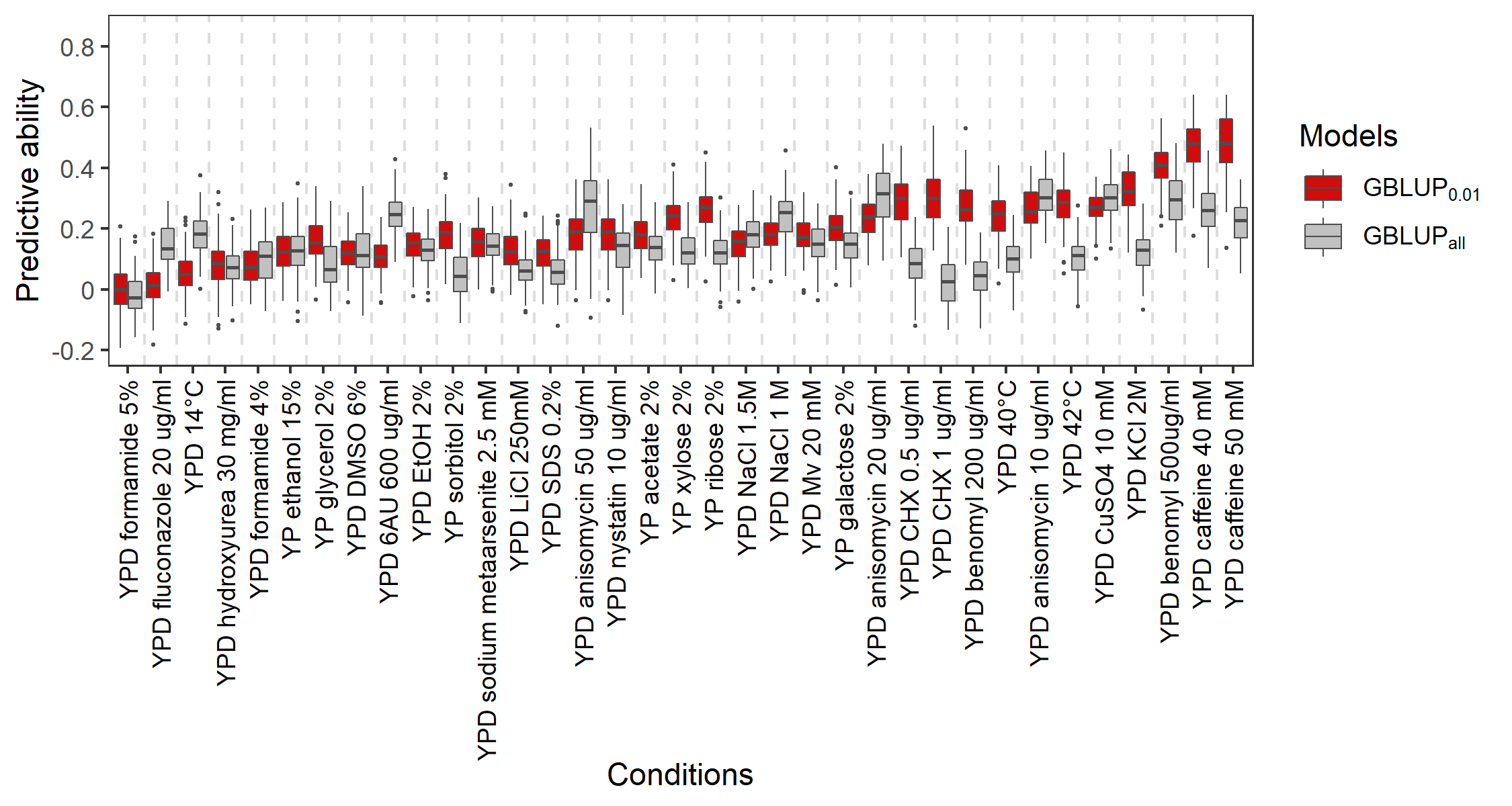

Supplement: S2 Fig — GBLUPall using all SNPs, and GBLUP0.01 using SNPs with MAF ≥ 0.01. (TIF) [file pgen.1008995.s002.tif]

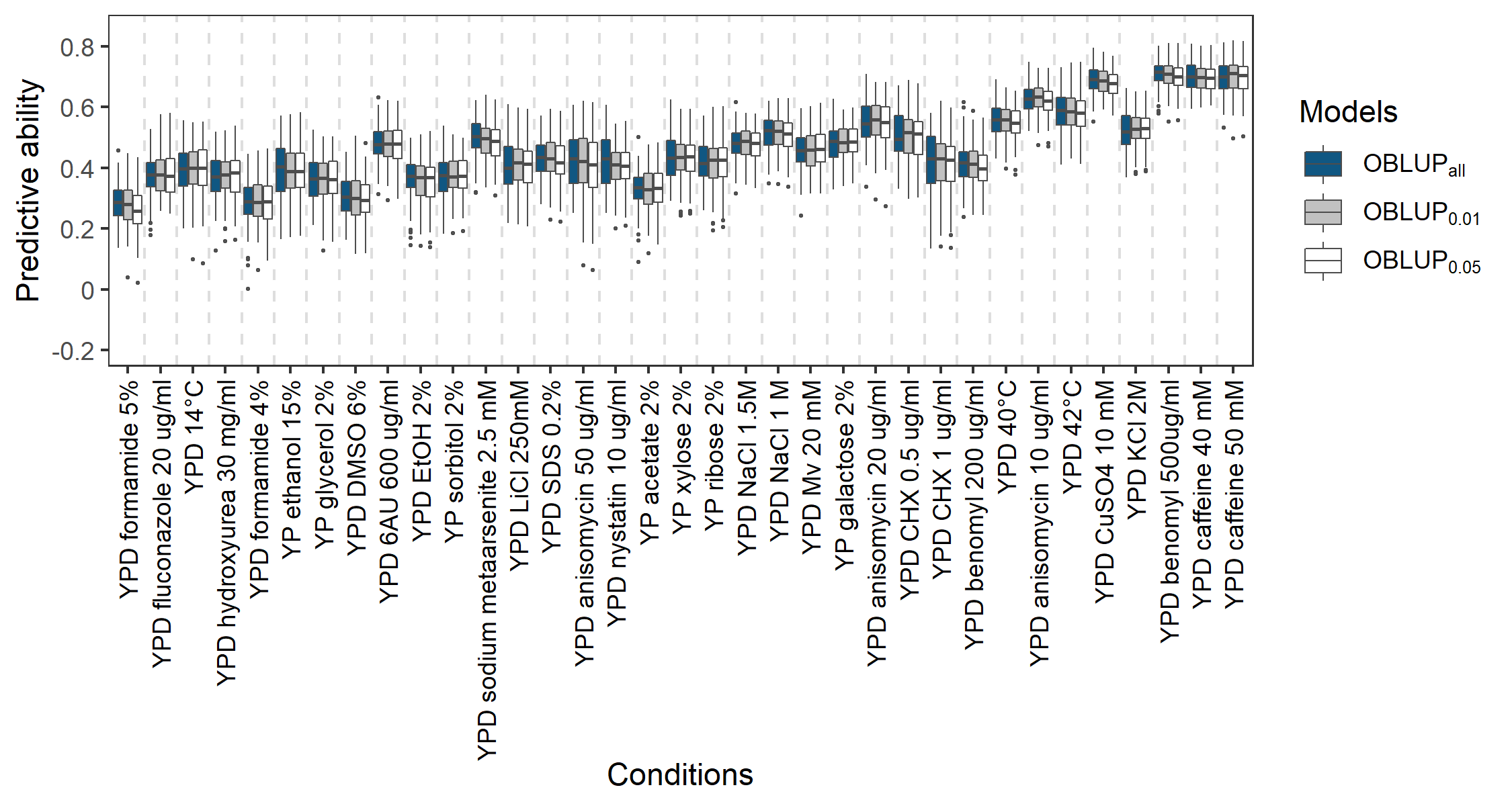

Supplement: S3 Fig — OBLUPall using all ORFs, OBLUP0.01 using ORFs with frequency ≥ 0.01, and OBLUP0.05 using ORFs with frequency ≥ 0.05, respectively. (TIF) [file pgen.1008995.s003.tif]

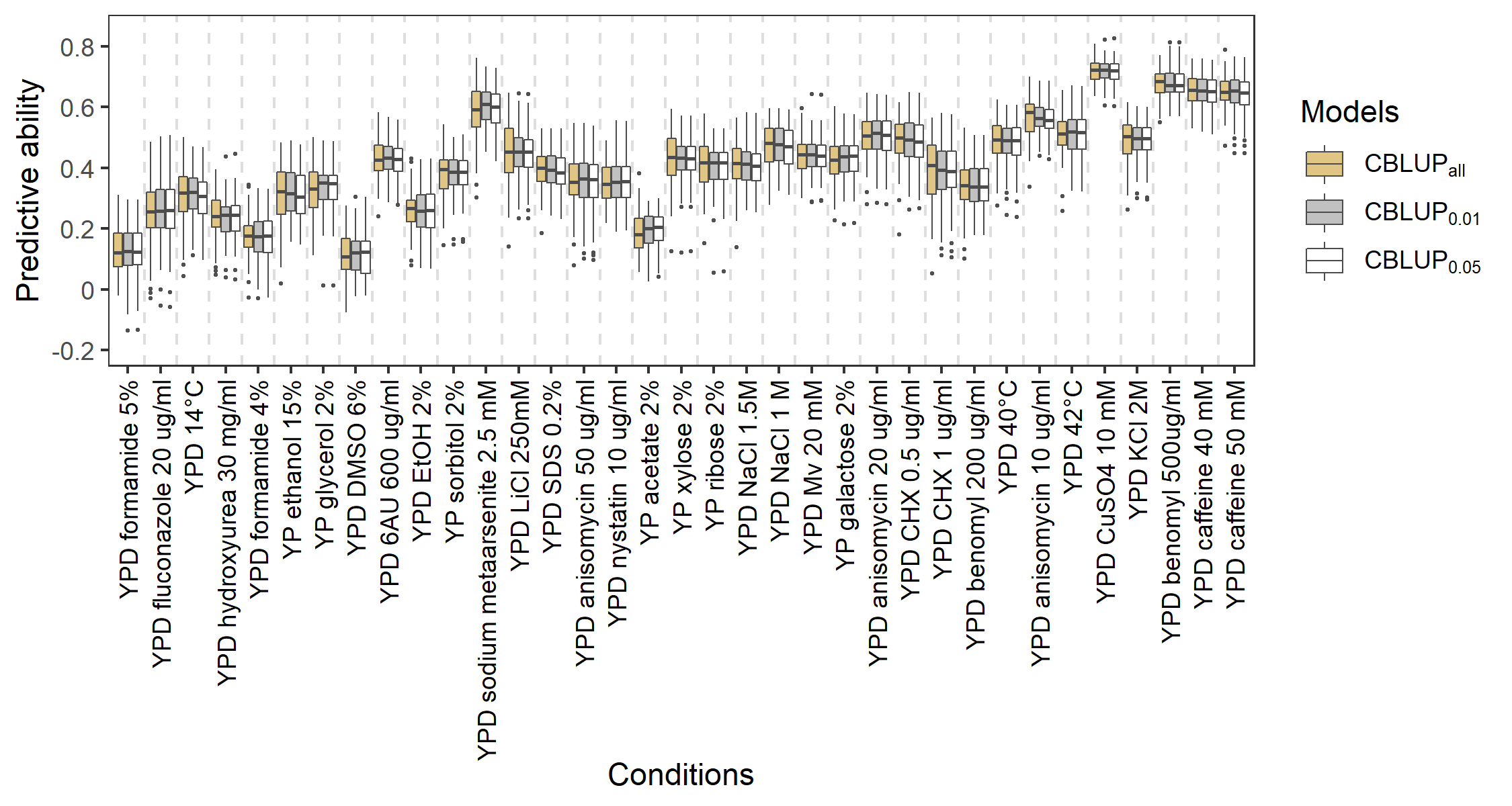

Supplement: S4 Fig — CBLUPall using all CNOs, CBLUP0.01 using CNOs with frequency ≥ 0.01, and CBLUP0.05 usig CNOs with frequency ≥ 0.05, respectively. (TIF) [file pgen.1008995.s004.tif]

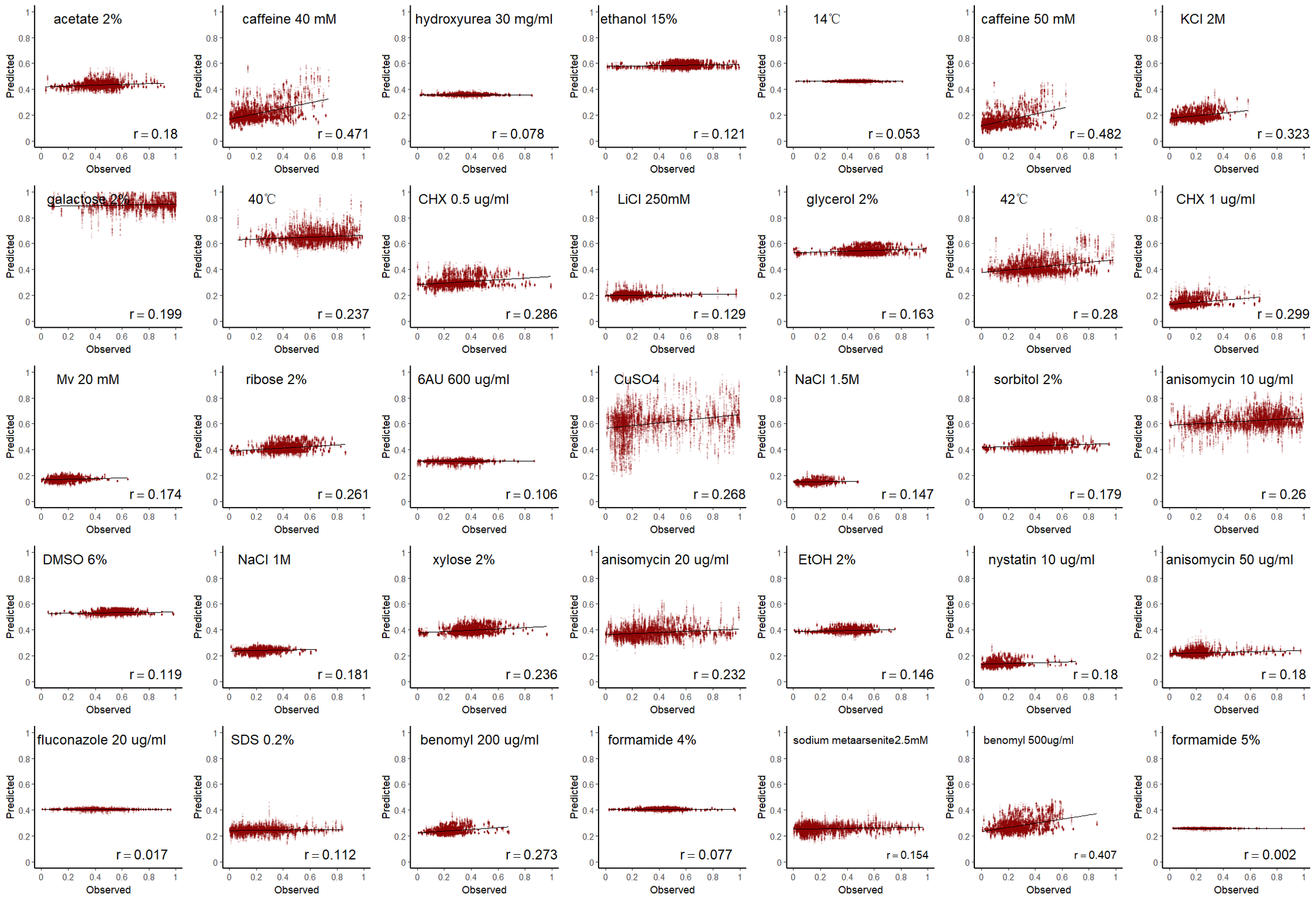

Supplement: S5 Fig — r represents the correlation coefficient between predicted and observed phenotypes across 35 traits. (TIF) [file pgen.1008995.s005.tif]

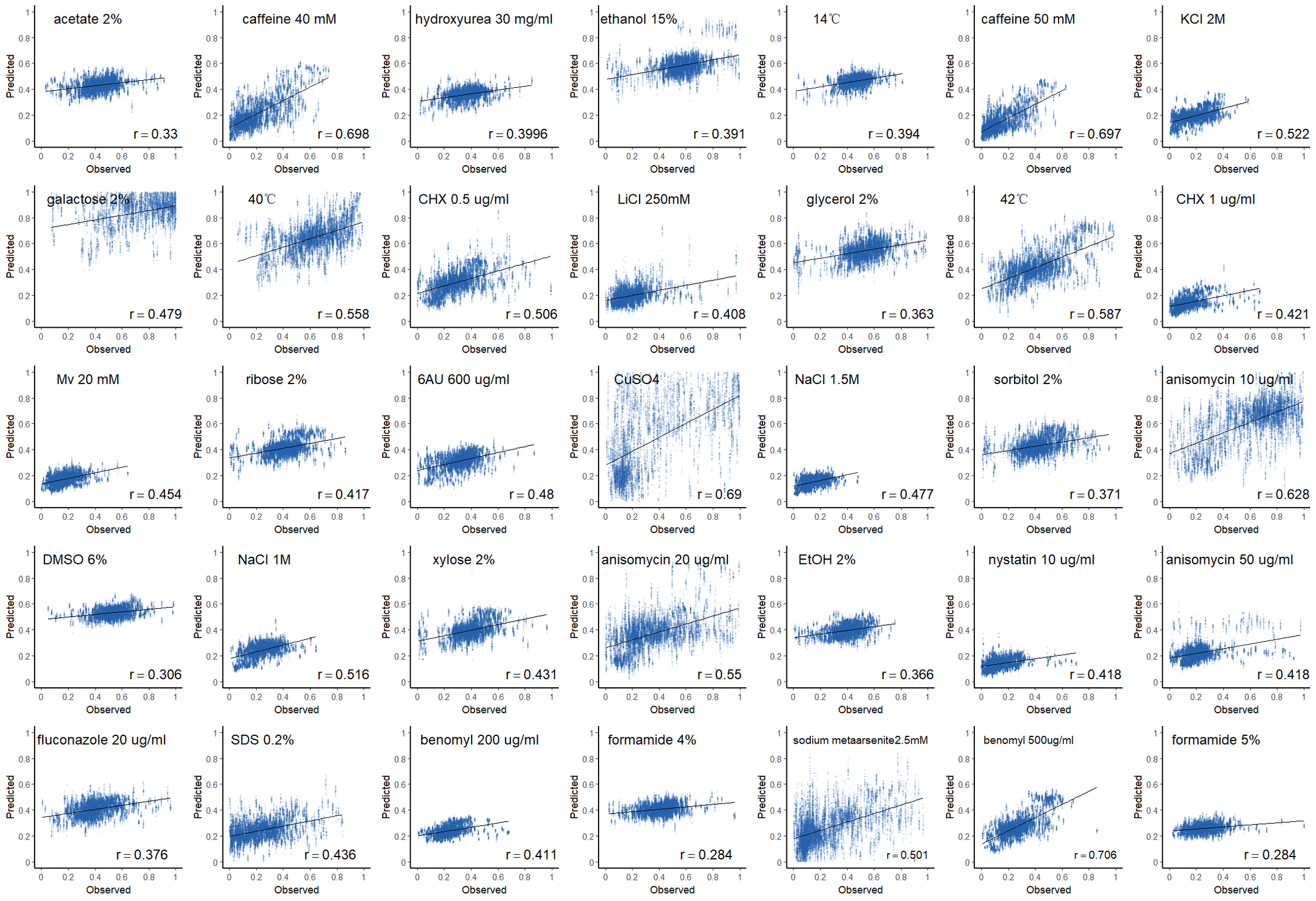

Supplement: S6 Fig — r represents the correlation coefficient between predicted and observed phenotypes across 35 traits. (TIF) [file pgen.1008995.s006.tif]

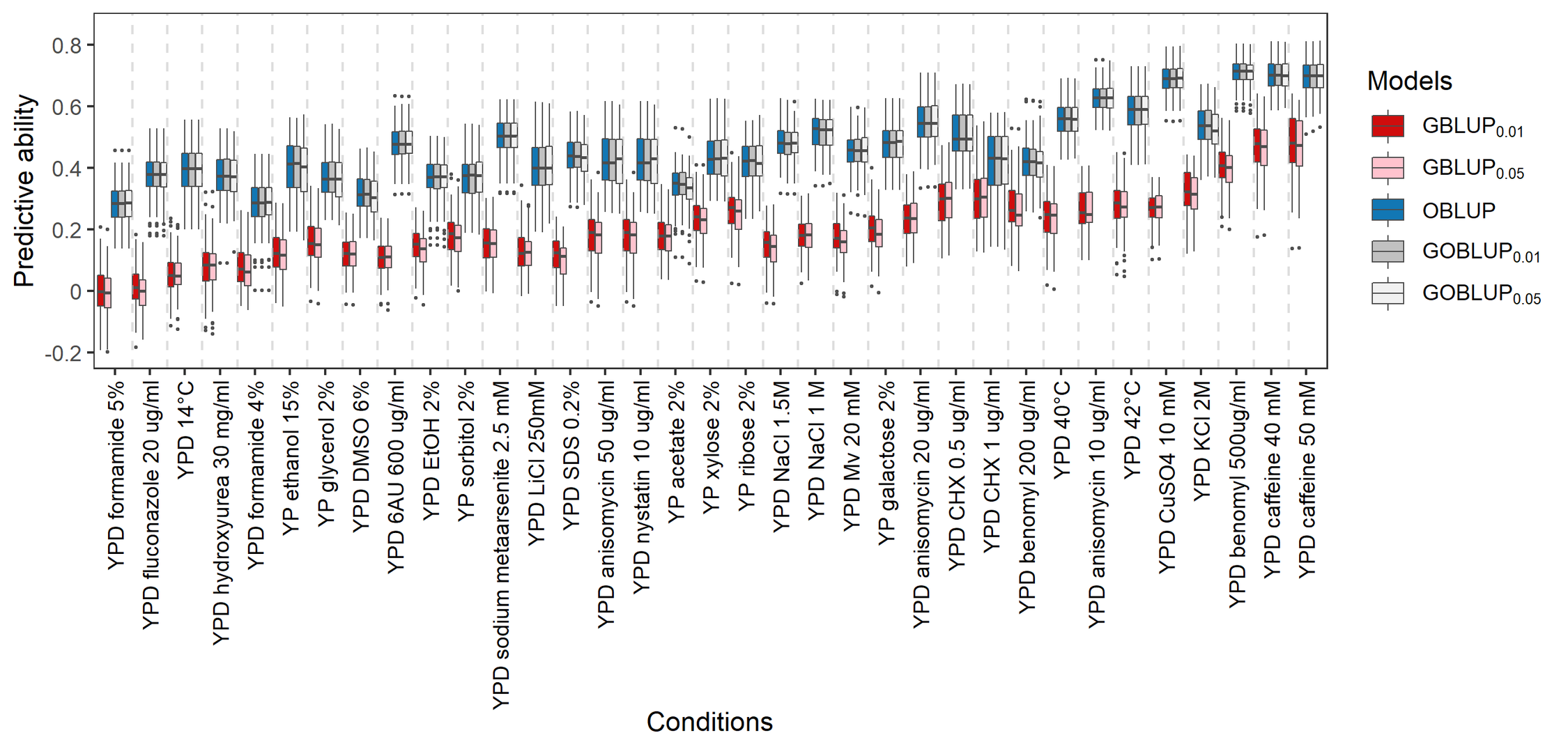

Supplement: S7 Fig — GBLUP0.01 using SNPs with MAF ≥ 0.01, GBLUP0.05 using SNPs with MAF ≥ 0.05, OBLUP using pan-genomic open reading frames, GOBLUP0.01 using both SNPs with MAF ≥ 0.01 and pan-genomic open reading frames, GOBLUP0.05 using both SNPs with MAF ≥ 0.05 and pan-genomic open reading frames. (TIF) [file pgen.1008995.s007.tif]

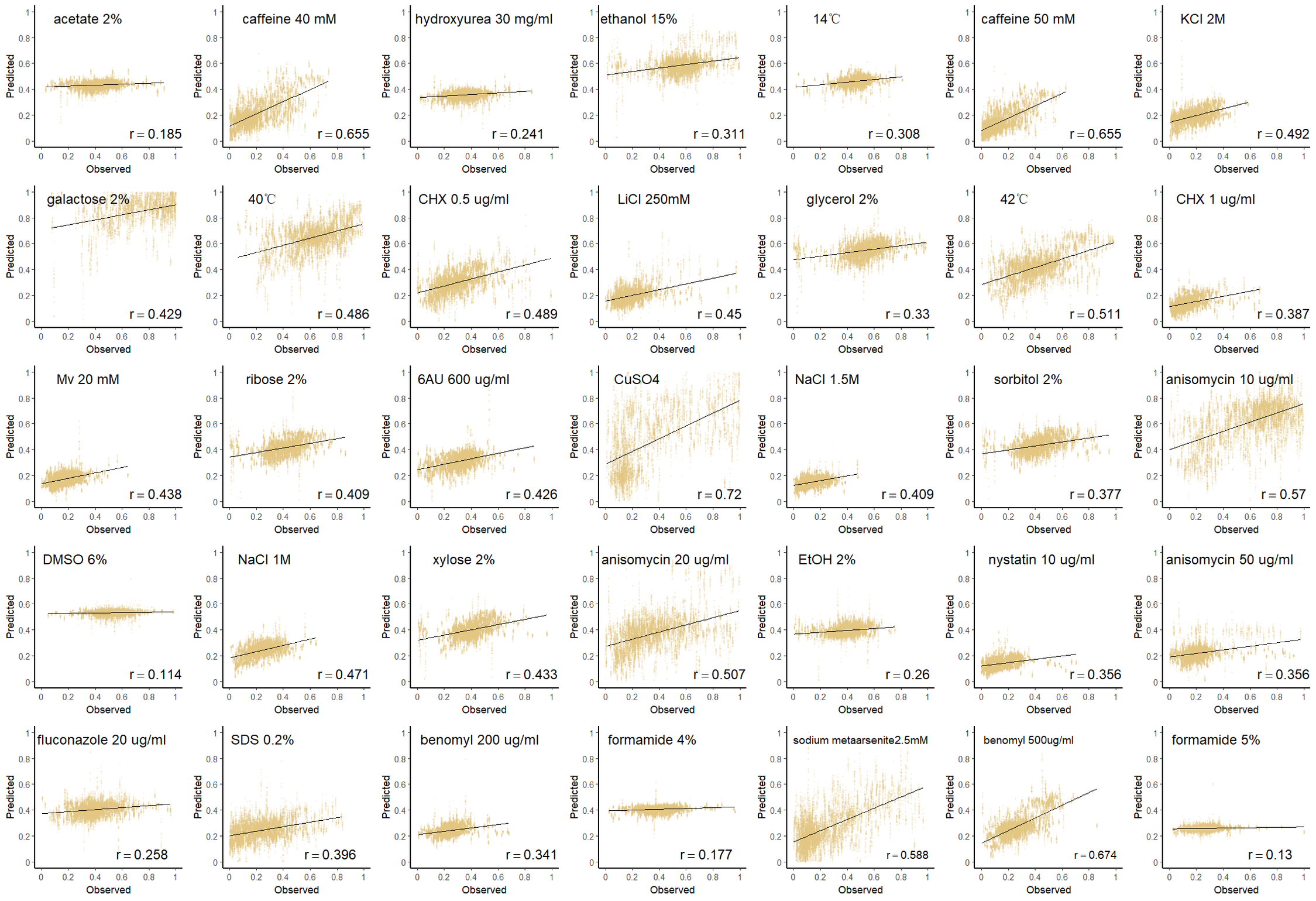

Supplement: S8 Fig — r represents the correlation coefficient between predicted and observed phenotypes across 35 traits. (TIF) [file pgen.1008995.s008.tif]

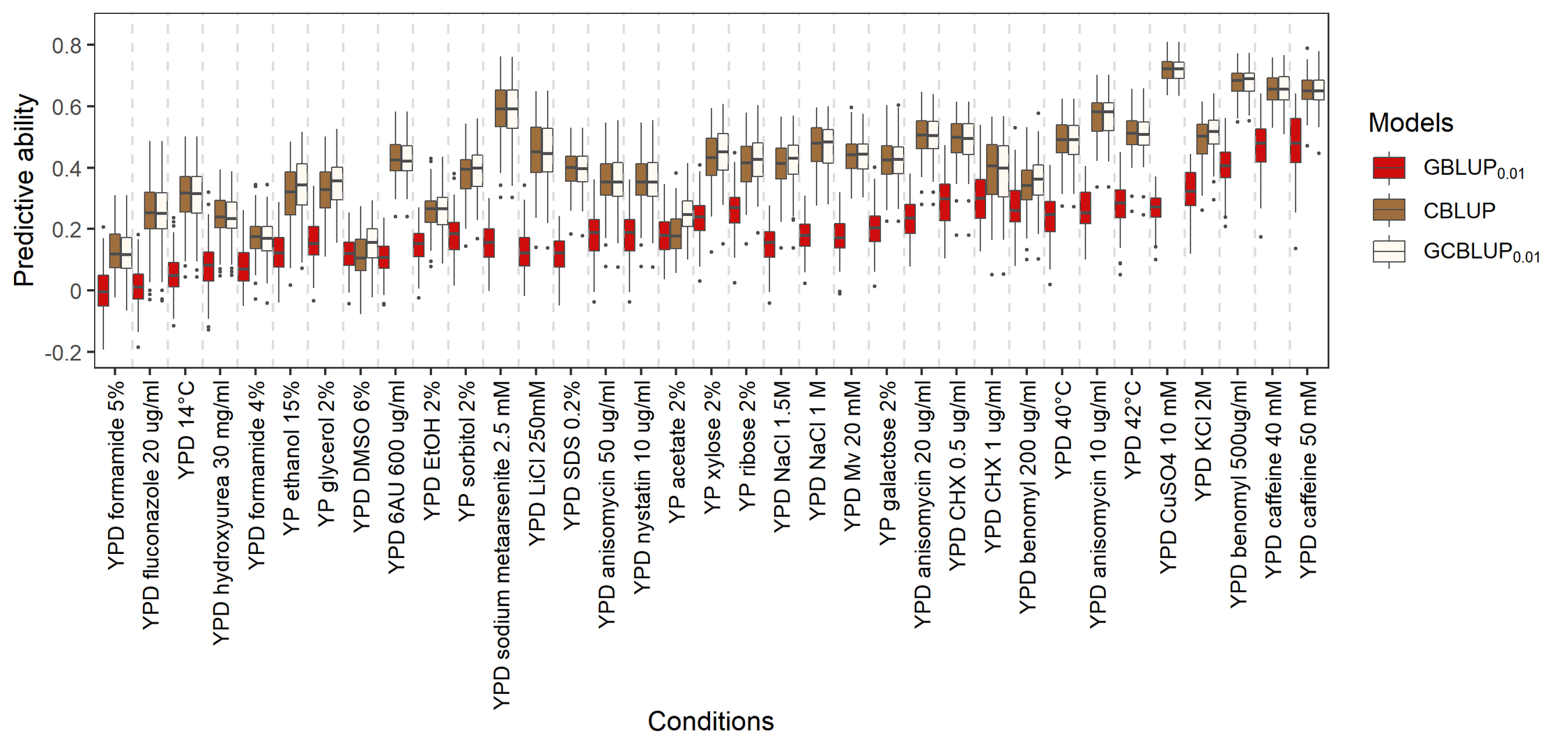

Supplement: S9 Fig — GBLUP0.01 using SNPs with MAF ≥ 0.01, CBLUP using copy numbers of pan-genomic open reading frames, GCBLUP0.01 using both SNPs with MAF ≥ 0.01 and copy numbers of pan-genomic open reading frames. (TIF) [file pgen.1008995.s009.tif]

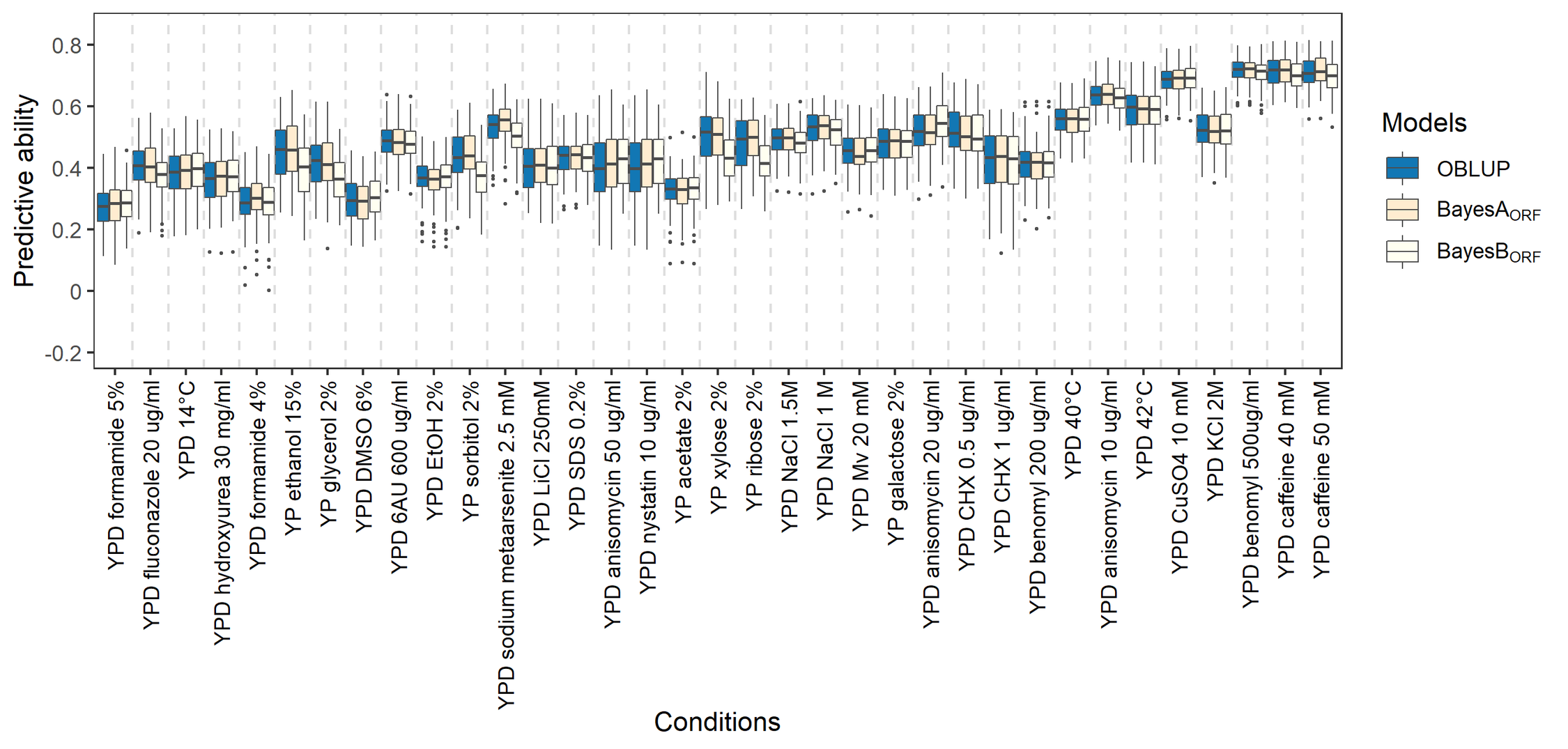

Supplement: S10 Fig — (TIF) [file pgen.1008995.s010.tif]

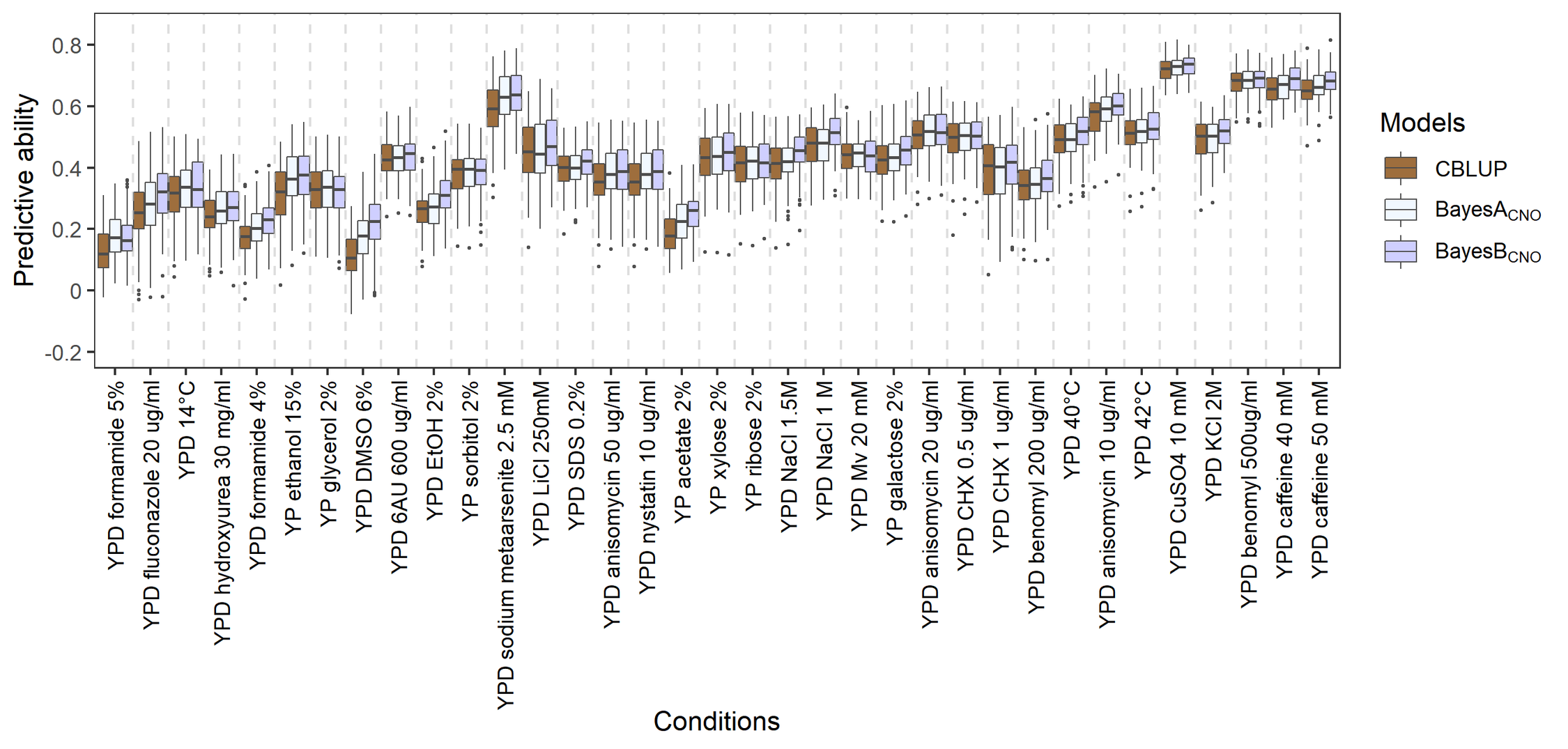

Supplement: S11 Fig — (TIF) [file pgen.1008995.s011.tif]

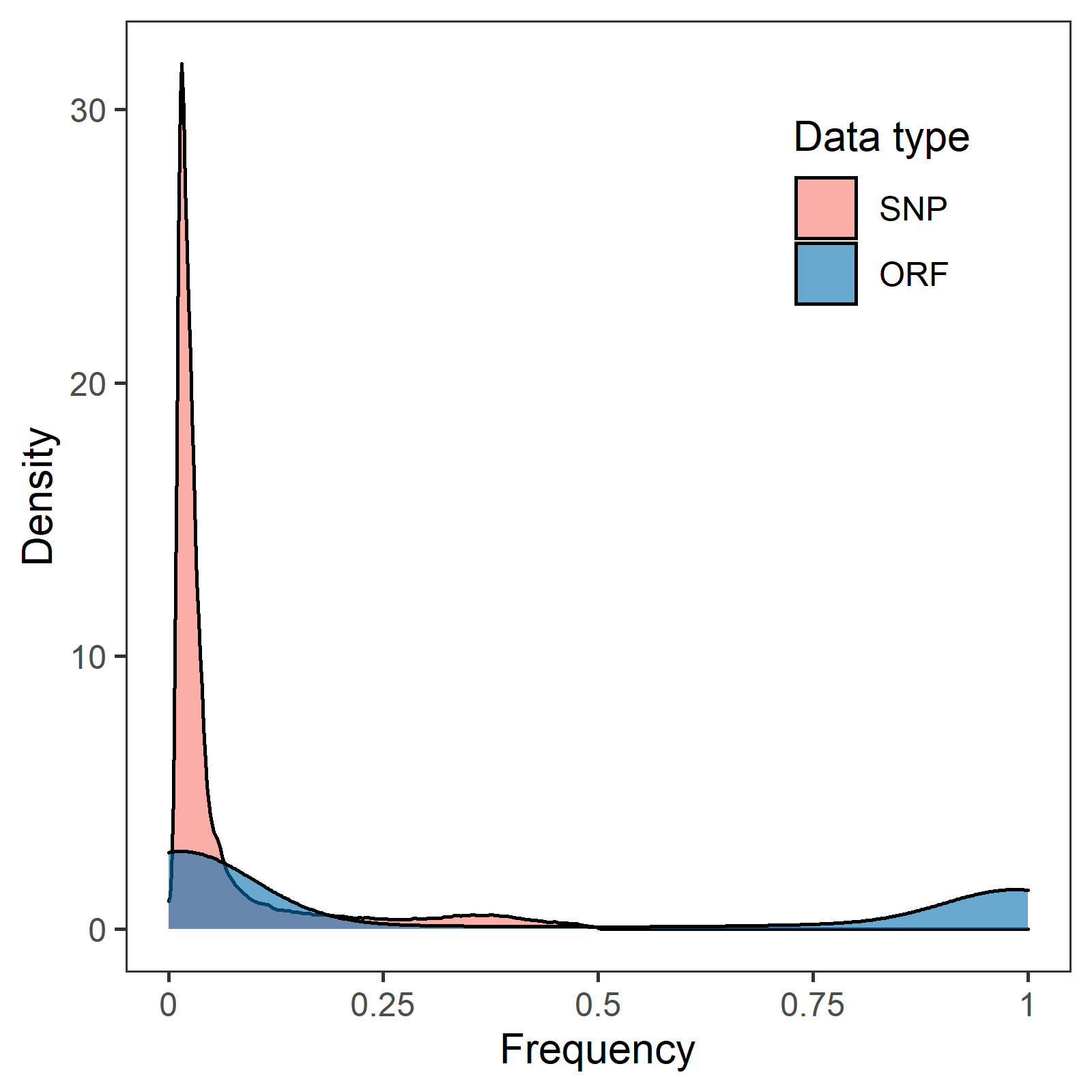

Supplement: S12 Fig — (TIF) [file pgen.1008995.s012.tif]
