# Supplementary material for: Pan-genomic open reading frames: A potential supplement of single nucleotide polymorphisms in estimation of heritability and genomic prediction
Source: PLoS Genet. 2020 Aug 24;16(8):e1008995. doi: 10.1371/journal.pgen.1008995 (PMC7470747; doi:10.1371/journal.pgen.1008995)
Supplement: S1 Table — GBLUP, OBLUP, CBLUP, GOBLUP and GCBLUP. h^G2 denoted the SNP-based heritability; h^O2 the ORF-based heritability; h^C2 the CNO-based heritability; h^GO2 the SNP-ORF-based heritability; h^GC2 the SNP-CNO-based heritability. (PDF) [file pgen.1008995.s013.pdf]

Conditions	$\hat{h}^2_G$	$\hat{h}^2_O$	$\hat{h}^2_C$	$\hat{h}^2_{GO}$	$\hat{h}^2_{GC}$
YPD formamide 5%	0.004 ± 0.002	0.634 ± 0.004	0.445 ± 0.019	0.636 ± 0.003	0.447 ± 0.021
YPD fluconazole 20 ug/ml	0.019 ± 0.003	0.710 ± 0.003	0.966 ± 0.003	0.708 ± 0.003	0.966 ± 0.003
YPD 14°C	0.021 ± 0.003	0.787 ± 0.002	0.965 ± 0.001	0.788 ± 0.002	0.962 ± 0.001
YPD hydroxyurea 30 mg/ml	0.027 ± 0.001	0.766 ± 0.002	0.9 ± 0.003	0.766 ± 0.002	0.91 ± 0.003
YPD formamide 4%	0.032 ± 0.003	0.698 ± 0.004	0.638 ± 0.01	0.698 ± 0.004	0.63 ± 0.01
YP ethanol 15%	0.059 ± 0.002	0.711 ± 0.004	0.971 ± 0.001	0.713 ± 0.004	0.973 ± 0.003
YP glycerol 2%	0.094 ± 0.003	0.674 ± 0.004	0.957 ± 0.001	0.671 ± 0.002	0.955 ± 0.001
YPD DMSO 6%	0.104 ± 0.004	0.655 ± 0.004	0.44 ± 0.019	0.656 ± 0.003	0.441 ± 0.021
YPD 6AU 600 ug/ml	0.094 ± 0.005	0.745 ± 0.002	0.98 ± 0	0.746 ± 0.002	0.981 ± 0.003
YPD EtOH 2%	0.165 ± 0.004	0.730 ± 0.002	0.96 ± 0.001	0.732 ± 0.002	0.963 ± 0.001
YP sorbitol 2%	0.162 ± 0.005	0.761 ± 0.003	0.976 ± 0	0.761 ± 0.003	0.972 ± 0
YPD sodium metaarsenite 2.5 mM	0.181 ± 0.006	0.900 ± 0.001	0.999 ± 0	0.89± 0.001	0.993 ± 0.005
YPD LiCl 250mM	0.184 ± 0.006	0.823 ± 0.002	0.994 ± 0	0.823 ± 0.002	0.993 ± 0
YPD SDS 0.2%	0.219 ± 0.011	0.786 ± 0.002	0.988 ± 0	0.787 ± 0.002	0.987 ± 0
YPD anisomycin 50 ug/ml	0.226 ± 0.007	0.668 ± 0.003	0.974 ± 0.001	0.667 ± 0.003	0.976 ± 0.001
YPD nystatin 10 ug/ml	0.312 ± 0.007	0.717 ± 0.003	0.955 ± 0.001	0.718 ± 0.001	0.956 ± 0.003
YP acetate 2%	0.232 ± 0.005	0.672 ± 0.003	0.829 ± 0.006	0.672 ± 0.003	0827± 0.006
YP xylose 2%	0.258 ± 0.006	0.768 ± 0.003	0.985 ± 0	0.768 ± 0.003	0.985 ± 0.001
YP ribose 2%	0.266 ± 0.005	0.727 ± 0.003	0.983 ± 0	0.726 ± 0.002	0.981 ± 0
YPD NaCl 1.5M	0.279 ± 0.009	0.778 ± 0.002	0.989 ± 0	0.778 ± 0.002	0.985 ± 0.001
YPD NaCl 1 M	0.268 ± 0.005	0.844 ± 0.001	0.995 ± 0	0.843 ± 0.002	0.991 ± 0
YPD Mv 20 mM	0.272 ± 0.006	0.796 ± 0.002	0.989 ± 0	0.795 ± 0.001	0.985 ± 0.002
YP galactose 2%	0.349 ± 0.007	0.739 ± 0.002	0.978 ± 0.001	0.739 ± 0.002	0.979 ± 0.001
YPD anisomycin 20 ug/ml	0.346 ± 0.006	0.823 ± 0.001	0.993 ± 0	0.824 ± 0.001	0.9943 ± 0
YPD CHX 0.5 ug/ml	0.384 ± 0.004	0.800± 0.002	0.992 ± 0	0.802 ± 0.002	0.992 ± 0.003
YPD CHX 1 ug/ml	0.419 ± 0.007	0.668 ± 0.003	0.983 ± 0.001	0.669± 0.004	0.986 ± 0.001
YPD benomyl 200 ug/ml	0.419 ± 0.005	0.623 ± 0.003	0.973 ± 0	0.622 ± 0.002	0.977 ± 0
YPD 40°C	0.433 ± 0.005	0.829 ± 0.001	0.99 ± 0	0.828 ± 0.001	0.991 ± 0

YPD anisomycin 10 ug/ml	0.479 ± 0.006	0.870 ± 0.001	0.995 ± 0	0.871 ± 0.001	0.991 ± 0.001
YPD 42°C	0.517 ± 0.005	0.816 ± 0.001	0.986 ± 0	0.816 ± 0.001	0.982 ± 0
YPD CuSO4 10 mM	0.523 ± 0.005	0.833 ± 0.001	0.991 ± 0	0.833 ± 0.001	0.991 ± 0
YPD KCl 2M	0.563 ± 0.005	0.764 ± 0.001	0.992 ± 0	0.764 ± 0.002	0.99 ± 0.001
YPD benomyl 500ug/ml	0.599 ± 0.003	0.834 ± 0.001	0.996 ± 0	0.834 ± 0.001	0.991 ± 0
YPD caffeine 40 mM	0.656 ± 0.003	0.854 ± 0.001	0.996 ± 0	0.854 ± 0.001	0.992 ± 0
YPD caffeine 50 mM	0.67 ± 0.003	0.857 ± 0.001	0.996 ± 0	0.857 ± 0.001	0.997 ± 0
