# Supplementary material for: Pan-genomic open reading frames: A potential supplement of single nucleotide polymorphisms in estimation of heritability and genomic prediction
Source: PLoS Genet. 2020 Aug 24;16(8):e1008995. doi: 10.1371/journal.pgen.1008995 (PMC7470747; doi:10.1371/journal.pgen.1008995)
Supplement: S2 Table — GBLUP, OBLUP, CBLUP, GOBLUP and GCBLUP. (PDF) [file pgen.1008995.s014.pdf]

Conditions	GBLUP	OBLUP	CBLUP	GOBLUP	GCBLUP
YPD formamide 5%	0.002 ± 0.007	0.284 ± 0.006	0.13 ± 0.008	0.281 ± 0.007	0.126 ± 0.008
**YPD fluconazole 20 ug/ml**	0.017 ± 0.007	0.376 ± 0.007	0.258 ± 0.01	0.375 ± 0.007	0.257 ± 0.01
**YPD 14°C**	0.053 ± 0.007	0.394 ± 0.008	0.308 ± 0.009	0.394 ± 0.008	0.306 ± 0.009
**YPD hydroxyurea 30 mg/ml**	0.078 ± 0.008	0.369 ± 0.007	0.241 ± 0.007	0.373 ± 0.007	0.238 ± 0.007
YPD formamide 4%	0.077 ± 0.007	0.284 ± 0.008	0.177 ± 0.007	0.282 ± 0.008	0.172 ± 0.007
**YP ethanol 15%**	0.121 ± 0.007	0.391 ± 0.009	0.311 ± 0.009	0.402 ± 0.009	0.338 ± 0.009
**YP glycerol 2%**	0.163 ± 0.007	0.363 ± 0.008	0.33 ± 0.008	0.371 ± 0.007	0.353 ± 0.008
**YPD DMSO 6%**	0.119 ± 0.006	0.306 ± 0.007	0.114 ± 0.007	0.316 ± 0.007	0.152 ± 0.007
**YPD 6AU 600 ug/ml**	0.106 ± 0.005	0.48 ± 0.006	0.426 ± 0.006	0.479 ± 0.006	0.424 ± 0.006
**YPD EtOH 2%**	0.146 ± 0.006	0.366 ± 0.007	0.26 ± 0.007	0.367 ± 0.007	0.26 ± 0.007
YP sorbitol 2%	0.179 ± 0.007	0.371 ± 0.007	0.377 ± 0.008	0.371 ± 0.007	0.389 ± 0.008
YPD sodium metaarsenite 2.5 mM	0.154 ± 0.006	0.501 ± 0.007	0.588 ± 0.009	0.501 ± 0.007	0.586 ± 0.009
**YPD LiCl 250mM**	0.129 ± 0.007	0.408 ± 0.009	0.45 ± 0.009	0.407 ± 0.009	0.45 ± 0.009
**YPD SDS 0.2%**	0.112 ± 0.006	0.436 ± 0.006	0.396 ± 0.006	0.44 ± 0.007	0.392 ± 0.007
**YPD anisomycin 50 ug/ml**	0.18 ± 0.007	0.418 ± 0.009	0.356 ± 0.008	0.421 ± 0.009	0.355 ± 0.008
**YPD nystatin 10 ug/ml**	0.18 ± 0.007	0.418 ± 0.009	0.356 ± 0.008	0.421 ± 0.009	0.355 ± 0.008
**YP acetate 2%**	0.18 ± 0.006	0.33 ± 0.006	0.185 ± 0.007	0.346 ± 0.006	0.248 ± 0.006
**YP xylose 2%**	0.236 ± 0.007	0.431 ± 0.007	0.433 ± 0.008	0.432 ± 0.007	0.448 ± 0.008
**YP ribose 2%**	0.261 ± 0.007	0.417 ± 0.007	0.409 ± 0.008	0.421 ± 0.007	0.424 ± 0.007
**YPD NaCl 1.5M**	0.147 ± 0.006	0.477 ± 0.006	0.409 ± 0.008	0.48 ± 0.006	0.419 ± 0.008
**YPD NaCl 1 M**	0.181 ± 0.006	0.516 ± 0.006	0.471 ± 0.007	0.518 ± 0.006	0.47 ± 0.007
**YPD Mv 20 mM**	0.174 ± 0.006	0.454 ± 0.006	0.438 ± 0.006	0.456 ± 0.006	0.437 ± 0.006
**YP galactose 2%**	0.199 ± 0.007	0.479 ± 0.006	0.429 ± 0.008	0.478 ± 0.006	0.427 ± 0.007
**YPD anisomycin 20 ug/ml**	0.232 ± 0.007	0.55 ± 0.007	0.507 ± 0.007	0.548 ± 0.007	0.504 ± 0.007
**YPD CHX 0.5 ug/ml**	0.286 ± 0.008	0.506 ± 0.008	0.489 ± 0.007	0.505 ± 0.008	0.488 ± 0.007
**YPD CHX 1 ug/ml**	0.299 ± 0.008	0.421 ± 0.01	0.387 ± 0.011	0.419 ± 0.01	0.382 ± 0.011
**YPD benomyl 200 ug/ml**	0.273 ± 0.007	0.411 ± 0.007	0.341 ± 0.008	0.421 ± 0.007	0.356 ± 0.007
**YPD 40°C**	0.237 ± 0.008	0.558 ± 0.005	0.486 ± 0.007	0.558 ± 0.006	0.484 ± 0.007

**YPD anisomycin 10 ug/ml**	0.26 ± 0.007	0.628 ± 0.005	0.57 ± 0.006	0.627 ± 0.005	0.57 ± 0.006
**YPD 42°C**	0.28 ± 0.007	0.587 ± 0.006	0.511 ± 0.007	0.586 ± 0.006	0.508 ± 0.007
YPD CuSO4 10 mM	0.268 ± 0.005	0.69 ± 0.004	0.72 ± 0.004	0.689 ± 0.004	0.719 ± 0.004
**YPD KCl 2M**	0.323 ± 0.007	0.522 ± 0.006	0.492 ± 0.007	0.538 ± 0.006	0.512 ± 0.007
**YPD benomyl 500ug/ml**	0.407 ± 0.007	0.706 ± 0.004	0.674 ± 0.005	0.708 ± 0.004	0.677 ± 0.005
**YPD caffeine 40 mM**	0.471 ± 0.009	0.698 ± 0.005	0.655 ± 0.005	0.698 ± 0.005	0.654 ± 0.005
**YPD caffeine 50 mM**	0.482 ± 0.009	0.697 ± 0.005	0.655 ± 0.005	0.695 ± 0.005	0.654 ± 0.005
