# Supplementary material for: Pan-genomic open reading frames: A potential supplement of single nucleotide polymorphisms in estimation of heritability and genomic prediction
Source: PLoS Genet. 2020 Aug 24;16(8):e1008995. doi: 10.1371/journal.pgen.1008995 (PMC7470747; doi:10.1371/journal.pgen.1008995)
Supplement: S3 Table — (PDF) [file pgen.1008995.s015.pdf]

Conditions	Mean ± Standard error	Variance	Max value	Minimum value
YPD formamide 5%	0.258 ± 0.004	0.01	1	0.026
YPD fluconazole 20 ug/ml	0.405 ± 0.005	0.02	1.142	0.005
YPD 14°C	0.462 ± 0.003	0.008	0.81	0.021
YPD hydroxyurea 30 mg/ml	0.358 ± 0.004	0.01	0.849	0.02
YPD formamide 4%	0.406 ± 0.004	0.013	0.964	0.026
YP ethanol 15%	0.585 ± 0.006	0.033	1.517	0.013
YP glycerol 2%	0.546 ± 0.006	0.031	1.632	0.004
YPD DMSO 6%	0.533 ± 0.004	0.013	1.277	0.049
YPD 6AU 600 ug/ml	0.31 ± 0.004	0.015	1.213	0.002
YPD EtOH 2%	0.394 ± 0.004	0.011	0.756	0.008
YP sorbitol 2%	0.431 ± 0.005	0.021	1.455	0.01
YPD sodium metaarsenite 2.5 mM	0.256 ± 0.008	0.048	1.576	0.002
YPD LiCl 250mM	0.2 ± 0.004	0.014	0.971	0.003
YPD SDS 0.2%	0.244 ± 0.006	0.025	0.84	0.002
YPD anisomycin 50 ug/ml	0.222 ± 0.005	0.018	1.123	0.006
YPD nystatin 10 ug/ml	0.138 ± 0.003	0.009	0.705	0.001
YP acetate 2%	0.432 ± 0.004	0.016	1.125	0.034
YP xylose 2%	0.397 ± 0.004	0.016	1.345	0.01
YP ribose 2%	0.413 ± 0.005	0.016	1.113	0.006
YPD NaCl 1.5M	0.151 ± 0.003	0.005	0.48	0.003
YPD NaCl 1 M	0.241 ± 0.003	0.009	0.645	0.014
YPD Mv 20 mM	0.171 ± 0.003	0.007	0.641	0.002
YP galactose 2%	0.92 ± 0.01	0.082	1.903	0.075
YPD anisomycin 20 ug/ml	0.378 ± 0.009	0.061	1.464	0.003
YPD CHX 0.5 ug/ml	0.301 ± 0.005	0.023	1.135	0.001
YPD CHX 1 ug/ml	0.141 ± 0.004	0.01	1.132	0.002
YPD benomyl 200 ug/ml	0.239 ± 0.003	0.007	0.679	0.007
YPD 40°C	0.655 ± 0.007	0.044	1.318	0.066

YPD anisomycin 10 ug/ml	0.623 ± 0.009	0.067	1.318	0.001
YPD 42°C	0.418 ± 0.007	0.038	1.555	0.005
YPD $CuSO_4$ 10 mM	0.617 ± 0.016	0.2	1.756	0.01
YPD KCl 2M	0.194 ± 0.004	0.01	0.585	0.007
YPD benomyl 500ug/ml	0.278 ± 0.004	0.015	0.857	0.009
YPD caffeine 40 mM	0.211 ± 0.005	0.022	0.739	0.002
YPD caffeine 50 mM	0.15 ± 0.004	0.013	0.627	0.002
